# ProphTools: general prioritization tools for heterogeneous biological networks

**DOI:** 10.1093/gigascience/gix111

**Published:** 2017-11-24

**Authors:** Carmen Navarro, Victor Martínez, Armando Blanco, Carlos Cano

**Affiliations:** Department of Computer Science and Artificial Intelligence, University of Granada, Granada, Spain

**Keywords:** network analysis, prioritization, heterogeneous networks, long noncoding RNAs

## Abstract

**Background:**

Networks have been proven effective representations for the analysis of biological data. As such, there exist multiple methods to extract knowledge from biological networks. However, these approaches usually limit their scope to a single biological entity type of interest or they lack the flexibility to analyze user-defined data.

**Results:**

We developed ProphTools, a flexible open-source command-line tool that performs prioritization on a heterogeneous network. ProphTools prioritization combines a Flow Propagation algorithm similar to a Random Walk with Restarts and a weighted propagation method. A flexible model for the representation of a heterogeneous network allows the user to define a prioritization problem involving an arbitrary number of entity types and their interconnections. Furthermore, ProphTools provides functionality to perform cross-validation tests, allowing users to select the best network configuration for a given problem. ProphTools core prioritization methodology has already been proven effective in gene-disease prioritization and drug repositioning. Here we make ProphTools available to the scientific community as flexible, open-source software and perform a new proof-of-concept case study on long noncoding RNAs (lncRNAs) to disease prioritization.

**Conclusions:**

ProphTools is robust prioritization software that provides the flexibility not present in other state-of-the-art network analysis approaches, enabling researchers to perform prioritization tasks on any user-defined heterogeneous network. Furthermore, the application to lncRNA-disease prioritization shows that ProphTools can reach the performance levels of *ad hoc* prioritization tools without losing its generality.

## Findings

### Background

Biological processes are complex and usually involve a large amount of entities interacting with each other. In this sense, it has been proven that networks are an effective model to improve our understanding of such processes, and many methodologies that use a network representation to infer new hypotheses from existing biological knowledge have been made available in the recent years [1]. These approaches model biological entities as nodes in a graph, where weighted edges correspond to interactions or any type of relationship between the connected nodes or entities. Edge weight, in this sense, measures the strength of the represented relationship. Many approaches have been proposed to build biological networks from data sources and to perform inference tasks on them [2].

From protein-protein interaction prediction [3] to the identification of candidate disease genes to drug repositioning [4] or very recent applications on microbiology [5], it seems to be clear that inference on graph or network data structures can be effective for the purpose of finding relations between entities that interact in such ways [1]. These *in silico* predictions allow researchers to reduce the search space to focus on a small set of entities that are more likely to be related to the entities of interest.

Although there exist many bioinformatics graph analysis tools that are freely available, they present at least 1 of the following limitations.

The first limitation we encounter is that many of these recent approaches are limited to the analysis of features in a single homogeneous network; i.e., they consider 1 network of entities of the same type or domain (e.g., a protein-protein interaction network or a gene network). For instance, *RANKS* [6] performs node prioritization on some label or property by using kernelized score functions, taking into account both the global structure of the network and the neighborhood of the query nodes. Other approaches, like *SVD-phy*, try to find functional associations between genes based on their phylogenetic distributions [7]. Some approaches, such as *DRaWR* [8], widen the features included in a graph by allowing different types of relations between the nodes (i.e., different types of edges).

Other approaches like *FunRich* [9] increase the level of flexibility, allowing users to choose from different data sources to perform enrichment analysis, including the possibility of using a customized database.

On the other hand, there are approaches that allow inclusion of more than 1 network in the analysis or prioritization task, including different types of interacting entities. However, these methods are built *ad hoc* to solve a specific problem. Many of these approaches have been proposed for the identification of novel gene-disease potential associations [10] or drug-disease associations for drug repositioning [11]. These methods usually focus more on the data sources integrated into the network than on the algorithm used to propagate the information within and/or accross networks, or they provide an algorithm that is tightly coupled to the data sources in use. In this sense, they lack the possibility of adding new data sources to populate the networks or integrating additional networks with other biomedical entities. Furthermore, the application of these methods to new domains is very challenging, as software and data are tightly coupled.

Because biological analyses can include a wide range of interconnected entities, tools that are able to integrate knowledge from different entity types and sources of data in the form of networks are of interest. Furthermore, the continuous appearance of new data sources to choose from hampers the maintenance of an up-to-date database list.

ProphTools intends to tackle these problems by implementing a general and flexible open-source model for representing heterogeneous networks composed of an arbitrary number of entity types (subnetworks) to perform any user-defined prioritization. ProphTools is based on an approach that has been proven useful in several prioritization applications, such as gene-disease prioritization [12] and drug repositioning [13]. Nonetheless, this functionality has never been made available as general purpose software.

In this paper, we present ProphTools, an open-source, customizable tool that can be used for a wide range of prioritization applications. To illustrate this, we applied ProphTools to a prioritization case study on long noncoding RNAs (lncRNAs) and diseases and compared its peformance with recent *ad hoc* approaches proposed for this task. Further, the data to perform state-of-the-art drug repositioning and lncRNA-disease prioritization using ProphTools have also been made available [14,15].

### Approach

ProphTools methodology operates on a heterogeneous global graph, *G* = (*D, R*), where *D* is a set of entity subnetworks (nodes of the same biological type) and *R* is a set of relationship subnetworks (bipartite networks connecting 2 different types of nodes). Given a set of nodes *Q* from the query network *D_q_* and a target network *D_t_*, the goal prioritization task is to determine the degree of relationship of the nodes in *D_t_* to the query nodes in *Q*.

ProphTools performs this prioritization combining (i) a within-network propagation method similar to Flow Propagation that uses Random Walk with Restarts and (ii) a weighted across-network propagation [12]. As Algorithm 1 shows, these processes are repeatedly applied to each network in every path from the query network to the target network. Values propagated from the query network eventually reach the target network and are then compared with values propagated from the target nodes by correlation [16].

**Algorithm 1:** Prioritization from query subnet D_q_ to target subnet D_t_.

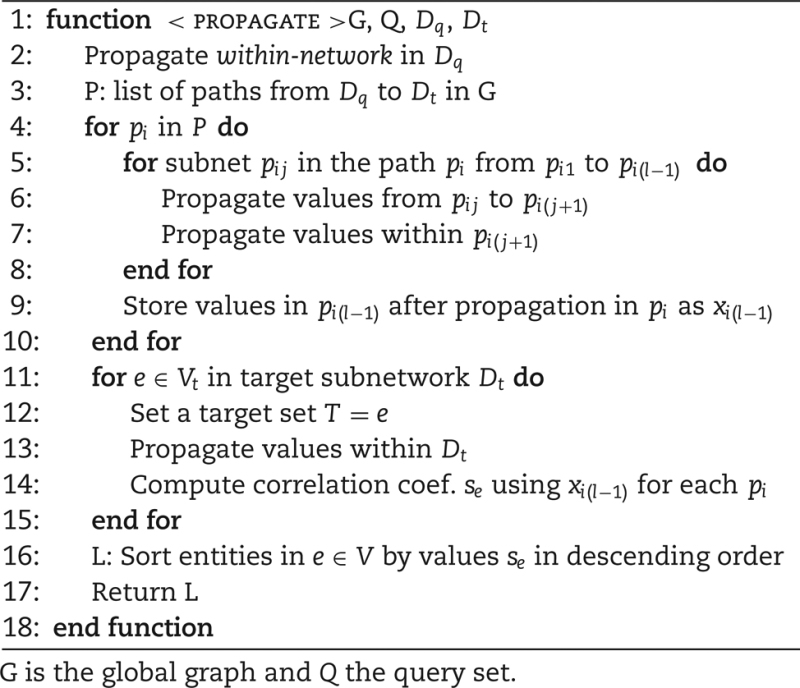

In addition, ProphTools can also run cross-validation (CV) tests to assess the performance of a given network configuration. For instance, a 5-fold CV test on such network configuration would remove one-fifth of the interactions connecting *D_q_* and *D_t_* and evaluate their predictability from the remaining network structure. The results are provided in the form of a receiver operating characteristic (ROC) curve, an area under the curve (AUC) value and a mean rank for each connection removed. These values can be used to compare the performance of different network configurations.

### Implementation

ProphTools is implemented in Python and does not require high computational resources, although memory requirements may increase with the size and density of the provided networks. The proposed package is built on broadly used python libraries that are freely available for download, such as NumPy high-performance array operation library, SciPy, and the scikit-learn Machine Learning library [17]. The core propagation method has been systematically tested using unit testing with a coverage of 86% for the entire package. In addition, Travis’s [18] platform for continuous integration has been connected with its repository in order to guarantee its successful deployment on a broad set of computers that meet its reduced software requirements.

Although ProphTools has been developed and tested natively in Linux, it relies on multiplatform libraries. ProphTools is available on GitHub as a Python package installable by pip [19]. In order to ensure that ProphTools can run on a wider set of computers, a Docker version has also been developed. ProphTools Docker version is freely available at DockerHub [20], allowing users of any operating system to easily run ProphTools as long as they have the Docker application installed.

Furthermore, ProphTools is open source and highly modular, allowing users to easily extend it with alternative propagation methods and scoring functions.

### How to use

**Figure 1.**
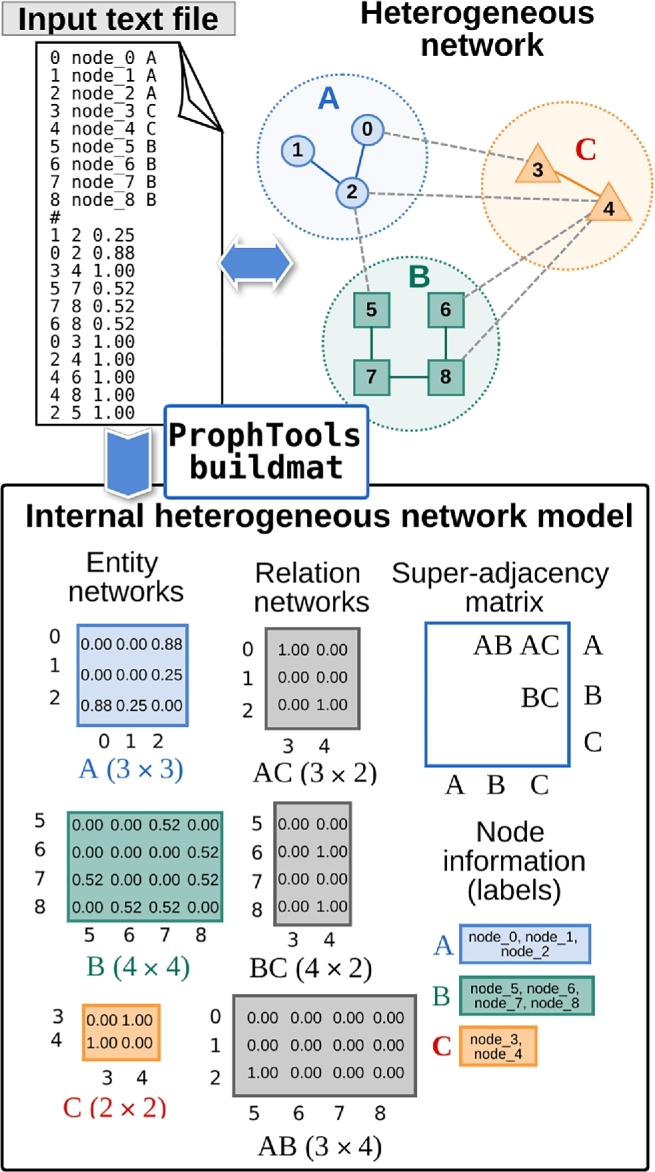
ProphTools heterogeneous network representation model is generated from an input text file provided by the user. This example shows a network with 3 types of entities: A, B, C. For each node, a numerical ID, a label, and type are specified. Edges, along with their weight, are also provided. ProphTools automatically (buildmat) converts this input to an internal representation model. For each subnetwork, an adjacency matrix is computed and normalized. Raw edge values are explicitly shown on the adjacency matrices. Additionally, connections between different entities are modeled as bipartite adjacency matrices. Finally, a super-adjacency matrix models how each relation matrix connects 2 entity matrices.

ProphTools uses internally a heterogeneous network representation file. A heterogeneous network is composed of (i) an arbitrary number of homogeneous subnetworks, each representing biological entities of the same type and their relations; and (ii) a set of bipartite subnetworks representing connections between entities of different types. A diagram showing the information included in this file can be seen in Fig. 1. This representation includes a weighted adjacency matrix for each subnetwork, a bipartite adjacency matrix for each subnetwork-subnetwork relation, and a super-adjacency matrix that provides information about which adjacency matrix corresponds to which entity relation. These matrix files can be built using the SciPy.sparse.io library, which is free and open source. Node labels are also included in the input file. This specific format is thoroughly explained in the ProphTools documentation.

This internal network specification allows ProphTools to perform prioritization tasks in any user-specified dataset. Nonetheless, in order to facilitate its application, ProphTools also supports 2 general network specification formats: a plain text format and Graph Exchange XML File (GEXF) file format [21].

The plain text format consists of a list of nodes and a list of edges, as in any regular graph, plus a label per node to specify which group each node belongs to. Figure 1 shows an example for a user-specified text file with 3 different subnetworks and its correspondence with the ProphTools internal heterogeneous network model.

Furthermore, the GEXF file format is based on XML, which is broadly used and flexible, allowing advanced users to ensure compatibility with other tools, such as the Gephi graph visualization tool [22].

ProphTools allows users to perform 2 operations: prioritization (run queries on specific sets of nodes for any of the networks) and performance tests (cross-validation).

For example, to apply ProphTools to the network configuration in Fig. 1, the user would first generate the ProphTools internal network model file:

prophtools buildmat —file example.txt —format txt —out example.mat

The user can perform any prioritization queries on the resulting mat file. For instance, to prioritize target nodes in subnetwork C from the query set *Q* = {0, 1} in subnetwork A:

prophtools prioritize —matfile example.mat —src A —dst C —qname 0,1 —out results.csv

To test the global performance of prioritizing target subnetwork C from query subnetwork A, the user could perform a 2-fold CV test:

prophtools cross —matfile example.mat —src A —dst C —fold 2 —out cvresults

Additional details and file examples are provided in ProphTools Git repository and documentation.

## Case Study: Long Noncoding RNA Disease Prioritization

Recent improvements in sequencing technology have proven that although less than 2% of the human genome codes for genes, more than 85% of the DNA is transcribed [23]. Whereas several types of these noncoding RNAs have been extensively studied, such as micro-RNAs and transfer RNAs [24], long noncoding RNAs (lncRNAs) are drawing an increasing interest in the recent years. A recent study estimates the amount of *loci* transcribing lncRNAs to be around 58 000 [25]. LncRNAs are, therefore, almost 3 times as abundant as coding genes according to our current knowledge of the human genome. However, little is known today about these biological entities, although it has been proven that lncRNAs play roles in cell regulation [26] and diseases [27].

Due to the increased relevance that long noncoding RNAs have acquired in the scientific community in the recent years, several *in silico* and *ad hoc* approaches have been published to systematically predict new relations between lncRNAs and diseases. *LncRNAdisease* [28] is a database including experimentally validated relations of lncRNAs and diseases and predictions based on these instances. *LRLSLDA* [28] defines a classification function based on the assumption that similarity between diseases can be an indicator of the similarity between the lncRNAs they are associated to. Later, their authors released *IRWRLDA* [29], a network-based lncRNA-disease prioritization algorithm that uses disease semantic similarity and lncRNA expression data to relate lncRNAs, and a modification of a Random Walk with Restarts (RWR) algorithm to perform prioritization. *RWRlncD* [30] also implements RWR on an lncRNA similarity network. LncRNA similarity is also based on the disease sets each lncRNA is associated to in the *lncRNAdisease* database [28]. A disadvantage of this method is that it can only perform prioritization on lncRNAs that are associated to at least 1 disease, which are a very small proportion (156) of the total number of lncRNAs annotated in the human genome (currently 15 787 lncRNA annotations in the latest release of GENCODE). More recently, Yao *et al.* proposed *LncPriCNet* [31], a method that built a multi-level network in order to perform lncRNA-disease prioritization.

All these approaches are *ad hoc* methods developed to solve the lncRNA-disease prioritization problem, not available as general source code. Additionally, the current lack of knowledge about lncRNAs and their relation to disease makes it probably difficult to draw conclusions about a broad set of lncRNAs, as available functional annotations are about 2 orders of magnitude smaller than the global amount of lncRNA candidates. Due to the interest these biological entities have drawn in recent years, it seems very likely that this knowledge will grow in the near future and more lncRNA-disease annotations will be made available. However, users will not be able to include future knowledge in these methods, as they are not available as flexible, general purpose tools.

**Figure 2.**
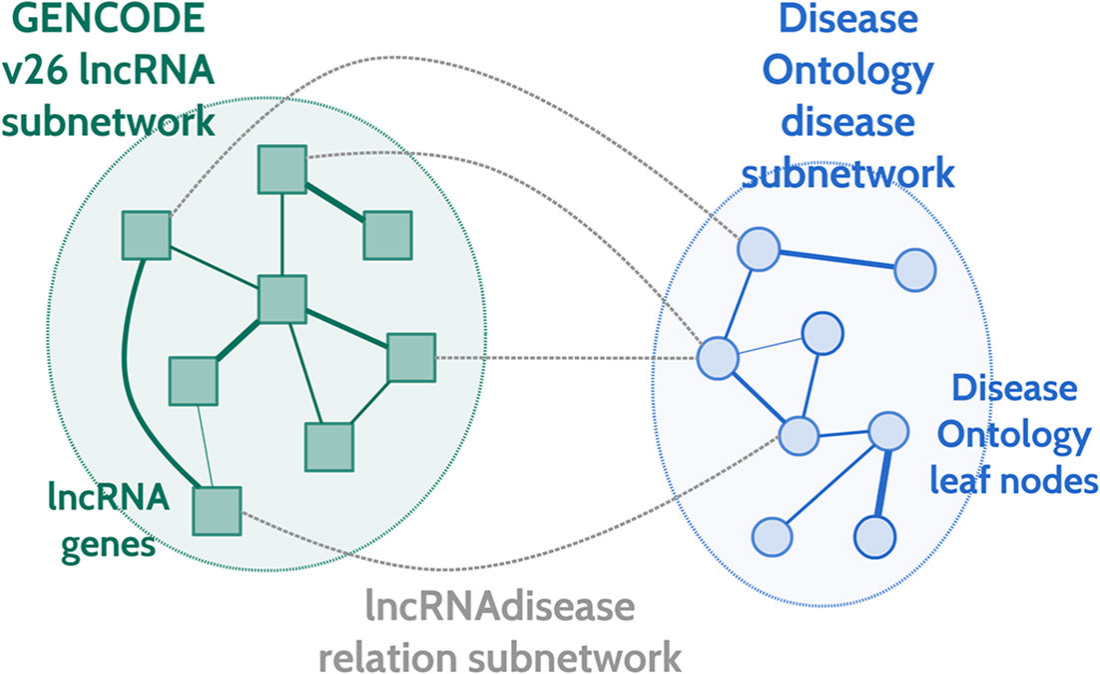
Heterogeneous network conguration built to perform lncRNA-disease ProphTools prioritization. LncRNA subnetwork is built from GENCODE v26 lncRNA sequences. Disease subnetwork is built from Disease Ontology leaf nodes using semantic similarity measures as in DrugNet [13]. LncRNA-disease relation subnetwork is taken from *lncRNAdisease* database [28]. This data configuration file is available at the ProphTools website.

Here, we apply ProphTools to lncRNA-disease prioritization as a proof of concept. To do so, we need to model this problem to fit the proposed heterogeneous network representation. Figure 2 shows the network configuration chosen to integrate the available data on lncRNAs and diseases.

Although ProphTools has not been specifically designed to accomplish this particular problem, obtained results are consistent with the current knowledge about lncRNAs, and ProphTools has proven as effective as other state-of-the art *ad hoc* methodologies. Furthermore, the datasets built are freely available to the scientific community to ensure reproducibility and allow further research and improvements on the topic.

### Data

The heterogeneous network includes 2 entity subnetworks: long noncoding RNAs (lncRNAs) and diseases, and a relation subnetwork lncRNA-disease connecting them (Fig. 2).

The lncRNA network was built using GENCODE v26 [32,33]. The 15 787 lncRNA gene annotations present in GENCODE v26 were processed by generating a projection of overlapping exons for each lncRNA and building a projected transcript representative of each lncRNA. The sequence of each projected transcript was then obtained from the repeat masked version of the human genome hg38. In order to reflect the modular functionality present in lncRNAs [34], we represented each lncRNA gene as a vector of hexamers (short subsequences 6 nucleotides in length). For each lncRNA gene sequence, the appearances of each of the 4096 possible hexamers were counted. These vectors were compared with each other to build an adjacency matrix using as a similarity measure the cosine similarity between the hexamer occurrence vectors. These similarities were used as edge weight in our lncRNA network. Additionally, the obtained adjacency matrix was postprocessed, removing 50% of the edges, in order to remove propagation noise while keeping the whole network as a single connected element. After this process, 125 isolated nodes (lncRNA genes) were removed from the final network, which connects 15 662 lncRNAs.

The disease network was obtained from the Disease Ontology, applying the same processing as described for Drugnet [13]. The resulting network includes 4517 diseases that correspond to leaf nodes in the Disease Ontology.

Finally, the lncRNA-disease network was built from the *lncRNAdisease* database [28]. A file corresponding to 1102 experimentally validated *lncRNA-disease* connections was downloaded from the *lncRNAdisease* website [35]. After removing duplicated connections in the lncRNA-disease file, 687 edges were obtained. Naming conventions used in this file for lncRNAs and diseases needed to be matched to GENCODE and the Disease Ontology identifiers, respectively. The ID matching process was performed using fuzzy string matching based on Levenshtein Distance [36], and results were manually revised. The lncRNA ID matching process resulted in a set of 229 matches out of 377, after removing general characterizations of sets of lncRNAs (e.g., RNA polymerase III-dependent lncRNAs) and filtering for human long noncoding RNAs. The disease matching process was performed using the same fuzzy string matching library, but 2 correspondence files were generated: 1 allowing multiple matches for each *lncRNAdisease* identifier and 1 storing only the best match. Because our disease network includes leaf nodes from the Disease Ontology, nonspecific identifiers such as “cancer” or “leukemia” correspond to a set of nodes in our disease network. The multiple matches approach thus includes even more redundancy in the network. To quantify and evaluate this effect in the results, we tested with both options.

After this identifier matching process, we obtained 2 lncRNA-disease datasets: (i) a generic dataset, consisting of 837 relations, including multiple synonyms for generic terms such as “cancer” (see Additional file 1), and (ii) a specific dataset, consisting of 352 relations where only the best match for the generic terms was included (see Aditional file 2). After removing connections to isolated lncRNA nodes, the resulting datasets have 829 and 347 relations, respectively.

### Results

**Figure 3.**
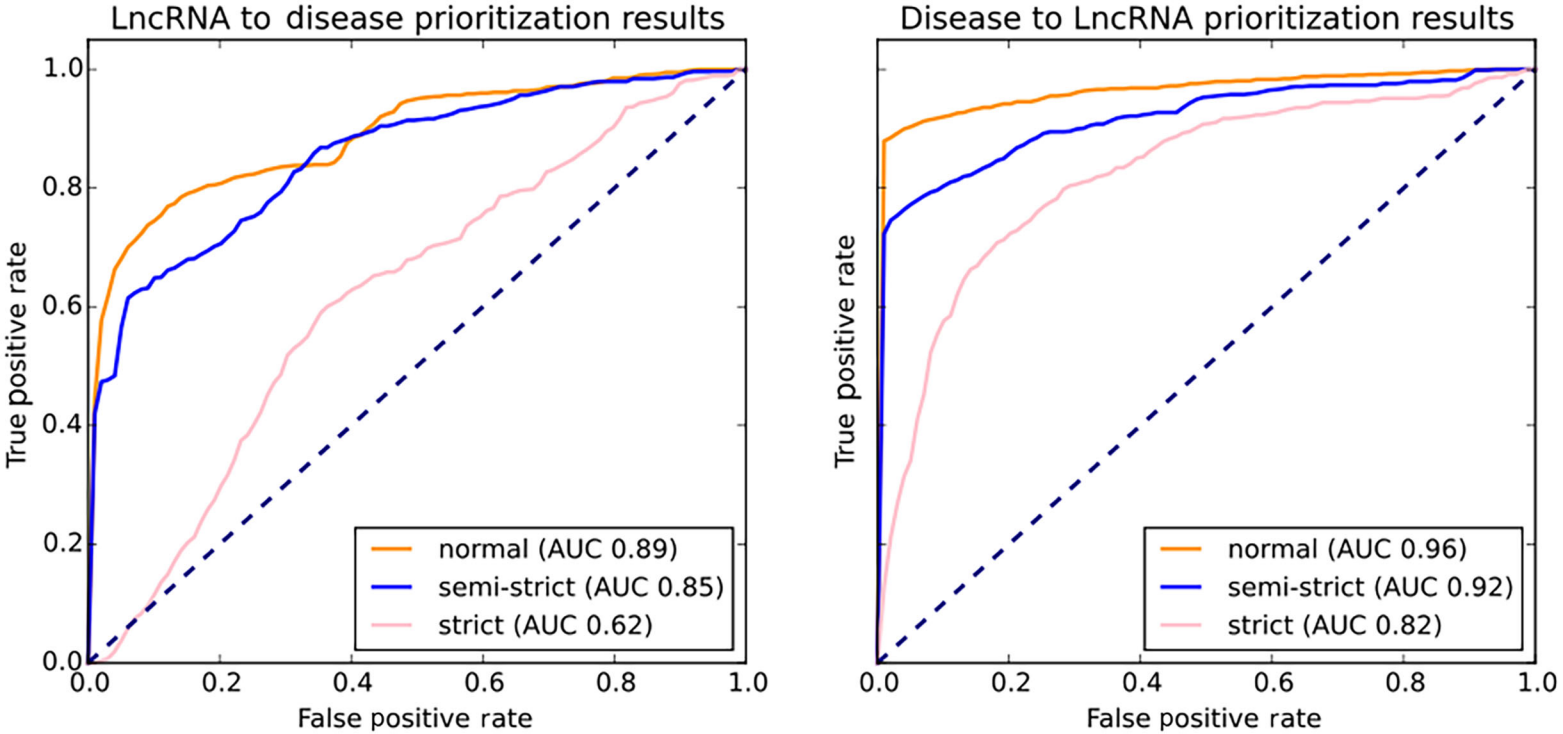
Results for 5-fold CV tests on the general dataset obtained from experimental evidence in the *LncRNAdisease* database. On the left side, ROC curves obtained for normal, semi-strict, and strict tests performed from lncRNA to disease. On the right side, ROC curves obtained for disease to lncRNA prioritization.

**Figure 4.**
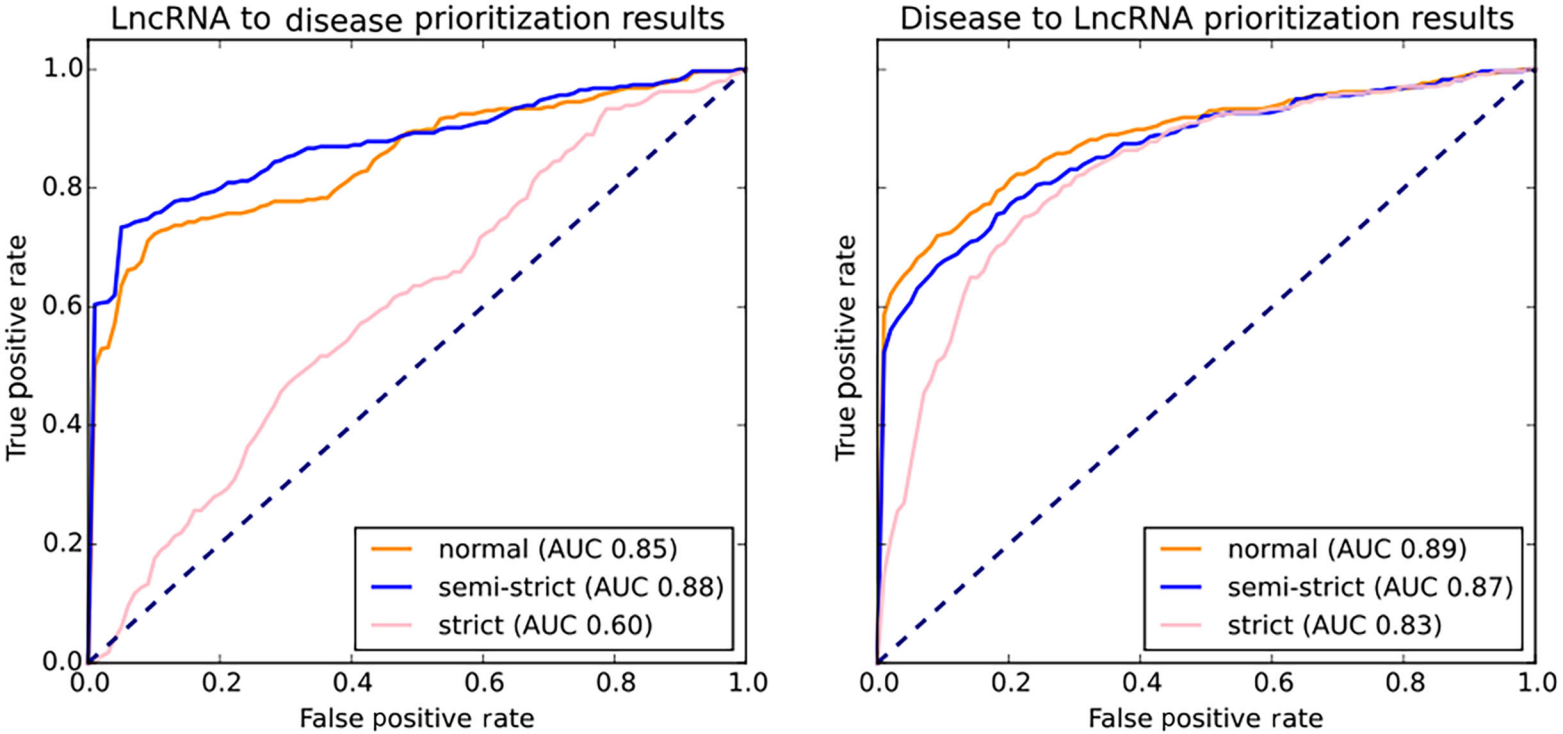
Results for 5-fold CV tests on the specific dataset obtained from experimental evidence in the *LncRNAdisease* database. On the left side, ROC curves obtained for the normal, semi-strict, and strict tests performed from lncRNA to disease. On the right side, ROC curves obtained for disease to lncRNA prioritization.

This heterogeneous network configuration was then tested for performance using 5-fold cross-validation. This functionality is also implemented in the ProphTools package. As our disease network includes leaf nodes from the Disease Ontology, nonspecific disease associations such as “cancer” or “leukemia” do not yield a single match, but a set of disease nodes. This results in a set of edges representing 1 correspondence in the original dataset, which could artificially improve the results in a performance test because it would allow a certain degree of redundancy that would persist after removing the tested edge. Taking this into account, we have performed 2 additional versions of the CV test, namely (i) a semi-strict version that removes at once all edges connecting a certain entity with the destination network in the direction of propagation and (ii) a strict version that removes all edges connecting the entities in both sides of the test edge (i.e., a semi-strict test for both directions of propagation). As Fig. 3 and 4 show, the AUC value is strongly affected by these 2 tests, especially if the direction of prioritization is lncRNA-disease (i.e., queries are lncRNAs and targets are diseases). Interestingly, if we perform a disease-lncRNA prioritization (queries are diseases and targets are lncRNAs), the semi-strict and strict CV tests have lower impact on the final results. This could be related to the Disease Ontology semantic similarity structure, which generates groups of strongly related nodes, such as families of diseases. Furthermore, the lncRNA network is scarcely populated. The amount of edges provided by the test datasets (829 general, 347 specific) is very reduced compared with the amount of diseases and lncRNAs in the lncRNA and disease networks, and there are groups of diseases, such as cancer, that cover a high percentage of the total dataset (∼23.66% for the general dataset and ∼18.18% for the specific dataset). Performing a strict test can eliminate not only the synonyms introduced in the general dataset, but also additional information that comes from a different source. If many of these cases occur, the resulting prioritization method tries to propagate from 1 network to another through very few connecting interactions, resulting in poor correlation scores. We believe this effect would be alleviated by a more populated lncRNA-disease network. However, it is interesting to note that although the strict test performs poorly for lncRNA-disease prioritization, the semi-strict test results are not affected by the removal of synonyms.

Normal, semi-strict, and strict results are reported in Table 1. Normal tests show a 0.963 ± 0.008 AUC value for lncRNA-disease prioritization and a 0.888 ± 0.015 AUC for disease-lncRNA prioritization for the general dataset (see Table 1), and a 0.850 ± 0.030 AUC value for lncRNA-disease prioritization and a 0.886 ± 0.012 AUC for disease-lncRNA prioritization for the specific dataset (see Table 2). These results show that predictions made by ProphTools with the proposed heterogeneous network configuration are consistent with current knowledge about lncRNAs and diseases and therefore likely to provide new predictions of interest. These AUC values are competitive with state-of-the art *ad hoc* approaches, such as *IRWRLDA* (0.7242 and 0.7872 AUC values) [29], *LRLSLDA* (0.7760 AUC value) [37] , and *RWRlncD* (0.822 AUC value) [30], and the recent *LncPriCNet* (0.93 AUC value) [31]. Furthermore, single prioritization queries on the specific dataset ran on average between 8.14 (±0.04) seconds for lncRNA-disease prioritization and 11.86 (±0.52) seconds for disease-lncRNA on our server (Intel(R) Xeon(R) CPU E5-2680 v3 @ 2.50GHz (×48), 256GiB RAM). The same test ran on average between 11.65 (±1.29) seconds for lncRNA-disease prioritization and 14.61 (±5.16) seconds for disease-lncRNA on a laptop.

**Table 1.** ProphTools performance results on the specific and general lncRNA-disease datasets for 3 different 5-fold CV modes

Dataset	CV test mode	Propagation direction	Mean AUC	Mean rank	Mean rank,%
General	Normal	lncRNA	0.963 ± 0.008	522.16 ± 120.21	3.33 ± 0.77
		disease	0.888 ± 0.015	503.67 ± 69.57	11.15 ± 1.54
	Semi-strict	lncRNA	0.917 ± 0.008	1240.95 ± 130.76	7.92 ± 0.83
		disease	0.854 ± 0.018	655.17 ± 78.45	14.50 ± 1.74
	Strict	lncRNA	0.823 ± 0.016	2767.30 ± 241.71	17.67 ± 0.01
		disease	0.618 ± 0.096	1725.18 ± 435.05	38.19 ± 9.63
Specific	Normal	lncRNA	0.886 ± 0.012	1751.24 ± 194.68	11.18 ± 1.24
		disease	0.850 ± 0.030	670.48 ± 135.53	14.84 ± 3.00
	Semi-strict	lncRNA	0.866 ± 0.026	2059.37 ± 403.71	13.15 ± 2.58
		disease	0.877 ± 0.020	548.05 ± 91.76	12.13 ± 2.03
	Strict	lncRNA	0.828 ± 0.034	2690.47 ± 476.54	17.18 ± 3.04
		disease	0.602 ± 0.058	1794.22 ± 261.58	39.72 ± 5.79

Normal CV removes only 1 edge per test. Semi-strict test mode removes all edges including origin nodes in the test set toward the propagation direction, and strict mode removes all edges involving the 2 nodes connected by each test edge in the test set. Propagation direction shows whether lncRNAs or diseases are are being ranked. Mean AUC column shows the average AUC obtained at the 5-fold CV test for each category. Mean rank shows the average ranking obtained for each test case, and Mean rank, %, shows the mean rank as percentage.

## Conclusions

ProphTools is an open-source, flexible, modular, and easy-to-use general implementation of a heterogeneous propagation algorithm that has been proven useful for relevant applications such as gene-disease prioritization and drug repositioning. The abstraction data layer we provide allows users to run ProphTools in any dataset of interest. As a proof of these features, a case study on lncRNA-disease prioritization has been described. Results are competitive with state-of-the art approaches in the field. In order to ensure the reproducibility of the results and allow further improvements in lncRNA-disease prioritization, the datasets built to apply ProphTools have also been made available.

ProphTools source code is available both as a GitHub repository and as a standalone Python package that can be easily installed via pip [19], and it also runs as a Docker container [20]. Additionally, ProphTools is not only open source but also very modular in design, allowing advanced users to extend its functionality. We are already working on more features (such as additional propagation algorithms) to incorporate into the ProphTools, framework in future versions. Although preprocessing work is required in order to build a network model, we believe ProphTools to be flexible representation of heterogeneous networks, and its support for different input file formats reduces the amount of work required to perform analyses in an *ad hoc* manner.

We expect that the availability of our prioritization method as an open-source, customizable tool can be of use for a wide range of biological applications.

## Availability of supporting source code and requirements

- Project name: ProphTools
- Project home page: https://github.com/cnluzon/prophtools, https://hub.docker.com/r/cnluzon/prophtools/
- Operating systems: Linux, platform independent if using the Docker version
- Programming language: Python 2.7
- Other requirements: Non-Linux systems need to run the Docker version. Native Linux systems require following Python libraries (installed automatically when installing via pip): NumPy (>=1.11.2), SciPy (>=0.18.1), matplotlib (>=1.4.3), scikit-learn (>=0.18), networkx (>=2.0).
- SciCrunch RRID (Research Resource Identification Initiative ID): SCR_015813
- License: GNU GPLv3.0

## Availability of supporting data and materials

ProphTools source code available at GitHub [19] and as a Docker container at Docker hub [20]. Heterogeneous network configurations for lncRNA-disease prioritization are available for download at the ProphTools website [15]. Drug-gene-disease prioritization data are also available at our server [14]. An archival copy of the code and other supporting data are available via the *GigaScience* repository, *Giga*DB [38].

## Abbreviations

AUC: area under the curve; CV: cross-validation; GEXF: Graph Exchange XML File; lncRNA: long noncoding RNA; ROC: receiver operating characteristic curve; RWR: Random Walk with Restarts.

## Competing interests

The authors declare that they have no competing interests.

## Funding

This work was supported by Dirección General de Investigación Científica y Técnica (TIN2013-41990-R and DPI2017-84439-R); European Regional Development Fund; and Spanish Ministry of Education, Culture and Sports (C. Navarro’s FPU grant).

## Author contributions

C.N. developed the software, both the Python repository and the Dockerized version, performed data analysis, and wrote the paper. V.M. developed the methodology and participated in the core software functionality. C.C. supervised the development of the software and data analysis, reviewed and edited the paper. A.B. conceptualized the research idea, supervised the quality of the process, and reviewed and edited the draft.

## Supplementary Material

GIGA-D-17-00123_Original-Submission.pdf

GIGA-D-17-00123_Revision-1.pdf

GIGA-D-17-00123_Revision-2.pdf

Response-to-Reviewer-Comments_Original-Submission.pdf

Response-to-Reviewer-Comments_Revision-1.pdf

Reviewer-1-Report-Original-Submission).pdf

Reviewer-2-Report-(Original-Submission).pdf

Reviewer-2-Report-(Revision-1).pdf

Supplemental material
