# Supplementary material for: ProphTools: general prioritization tools for heterogeneous biological networks
Source: Gigascience. 2017 Nov 24;6(12):1–8. doi: 10.1093/gigascience/gix111 (PMC5751048; doi:10.1093/gigascience/gix111)
Supplement: GIGA-D-17-00123_Original-Submission.pdf [file gix111_giga-d-17-00123_original-submission.pdf]

GigaScience

ProphTools: General Prioritization Tools for Heterogeneous Biological Networks

--Manuscript Draft--

Manuscript Number:	GIGA-D-17-00123
Full Title:	ProphTools: General Prioritization Tools for Heterogeneous Biological Networks
Article Type:	Technical Note
Funding Information:	Consejería de Economía, Innovación, Ciencia y Empleo, Junta de Andalucía (PI-0710-2013) — Dr. Armando Blanco Morón<br>Dirección General de Investigación Científica y Técnica (TIN2013-41990-R) — Dr. Armando Blanco Morón<br>European Regional Development Fund — Dr. Armando Blanco Morón<br>Ministerio de Educación, Cultura y Deporte (FPU12_01875) — Ms. Carmen Navarro Luzón
Abstract:	Background: Networks have been proven effective representations for the analysis of biological data. As such, there exist multiple methods to extract knowledge from biological networks. However, these approaches usually limit their scope to a single biological entity type of interest, or they lack flexibility to analyse user-defined data.<br>Findings: We developed ProphTools, a flexible open-source command-line tool that performs prioritization on a heterogeneous network. ProphTools prioritization combines a Flow Propagation algorithm similar to a Random Walk With Restarts and a weighted propagation method. A flexible model for the representation of a heterogeneous network allows the user to define a prioritization problem involving an arbitrary number of entity types and their interconnections. Furthermore, ProphTools provides functionality for performance Leave One Out with Cross-Validation tests, allowing users to select the best network configuration for any given problem. ProphTools core prioritization methodology has already been proven effective in gene-disease prioritization and drug repositioning. Here we make ProphTools available to the scientific community as flexible, open-source software and perform a new proof-of-concept case study on long non-coding RNAs (lncRNAs) to disease prioritization.<br>Conclusions: ProphTools is robust prioritization software that provides the flexibility not present in other state-of-the-art network analysis approaches, enabling researchers to perform prioritization tasks on any user-defined heterogeneous network. Furthermore, the application to lncRNA-disease prioritization showed that ProphTools can reach the performance levels of ad-hoc prioritization tools without losing generality.
Corresponding Author:	Carmen Navarro Luzón<br>Universidad de Granada<br>SPAIN
Corresponding Author Secondary Information:	
Corresponding Author's Institution:	Universidad de Granada
Corresponding Author's Secondary Institution:	
First Author:	Carmen Navarro Luzón
First Author Secondary Information:	
Order of Authors:	Carmen Navarro Luzón<br>Víctor Martínez Gómez<br>Armando Blanco Morón<br>Carlos Cano Gutiérrez
Order of Authors Secondary Information:	

Powered by Editorial Manager® and ProduXion Manager® from Aries Systems Corporation

**Opposed Reviewers:**	
**Additional Information:**	
**Question**	**Response**
Are you submitting this manuscript to a special series or article collection?	No
**Experimental design and statistics**<br><br>Full details of the experimental design and statistical methods used should be given in the Methods section, as detailed in our Minimum Standards Reporting Checklist. Information essential to interpreting the data presented should be made available in the figure legends.<br><br>Have you included all the information requested in your manuscript?	Yes
**Resources**<br><br>A description of all resources used, including antibodies, cell lines, animals and software tools, with enough information to allow them to be uniquely identified, should be included in the Methods section. Authors are strongly encouraged to cite Research Resource Identifiers (RRIDs) for antibodies, model organisms and tools, where possible.<br><br>Have you included the information requested as detailed in our Minimum Standards Reporting Checklist?	Yes
**Availability of data and materials**<br><br>All datasets and code on which the conclusions of the paper rely must be either included in your submission or deposited in publicly available repositories (where available and ethically appropriate), referencing such data using a unique identifier in the references and in the "Availability of Data and Materials" section of your manuscript.<br><br>Have you have met the above requirement as detailed in our Minimum Standards Reporting Checklist?	Yes

Powered by Editorial Manager® and ProduXion Manager® from Aries Systems Corporation

Manuscript
Click here to download Manuscript manuscript.pdf

MANUSCRIPT SUBMISSION

GIGASCIENCE JOURNAL

ProphTools: General Prioritization Tools for Heterogeneous Biological Networks

Authors:

Carmen NAVARRO
Department of Computer Science and Artificial Intelligence,
University of Granada, Spain.

Víctor MARTÍNEZ
Department of Computer Science and Artificial Intelligence,
University of Granada, Spain.

Armando BLANCO
Department of Computer Science and Artificial Intelligence,
University of Granada. Spain.

Carlos CANO
Department of Computer Science and Artificial Intelligence,
University of Granada, Spain.

Submitted: Friday 26th May, 2017

ProphTools: General Prioritization Tools for Heterogeneous Biological Networks*

Carmen Navarro[1], Víctor Martínez[1], Armando Blanco[1] and Carlos Cano[1]

[1]Department of Computer Science and Artificial Intelligence, University of Granada, Spain

Submitted: May 26th, 2017

Abstract

Background: *Networks have been proven effective representations for the analysis of biological data. As such, there exist multiple methods to extract knowledge from biological networks. However, these approaches usually limit their scope to a single biological entity type of interest, or they lack flexibility to analyse user-defined data.*

Findings: *We developed ProphTools, a flexible open-source command-line tool that performs prioritization on a heterogeneous network. ProphTools prioritization combines a Flow Propagation algorithm similar to a Random Walk With Restarts and a weighted propagation method. A flexible model for the representation of a heterogeneous network allows the user to define a prioritization problem involving an arbitrary number of entity types and their interconnections. Furthermore, ProphTools provides functionality for performance Leave One Out with Cross-Validation tests, allowing users to select the best network configuration for any given problem. ProphTools core prioritization methodology has already been proven effective in gene-disease prioritization and drug repositioning. Here we make ProphTools available to the scientific community as flexible, open-source software and perform a new proof-of-concept case study on long non-coding RNAs (lncRNAs) to disease prioritization.*

Conclusions: *ProphTools is robust prioritization software that provides the flexibility not present in other state-of-the-art network analysis approaches, enabling researchers to perform prioritization tasks on any user-defined heterogeneous network. Furthermore, the application to lncRNA-disease prioritization showed that ProphTools can reach the performance levels of ad-hoc prioritization tools without losing generality.*

Keywords— Network analysis, prioritization, heterogeneous networks, long non-coding RNAs

*Draft manuscript. Please do not cite without the authors' permission.

1 Findings

Background

Biological processes are complex and usually involve a large amount of entities interacting with each other. In this sense, it has been proven that networks are an effective model to improve our understanding of such processes, and many methodologies that use a network representation to infer new hypotheses from existing biological knowledge have been made available in the recent years [1]. These approaches model biological entities as nodes in a graph, whereas weighted edges correspond to interactions or any type of relationship between the connected nodes or entities. Edge weight, in this sense, measures the strength of the represented relationship. From protein-protein interaction prediction to the identification of candidate disease genes to drug repositioning, it seems to be clear that graph or network data structures are effective for the purpose of finding relations between entities that interact in such ways [1]. These *in-silico* predictions allow researchers to reduce the search space to focus on a small set of entities that are more likely to be related to the entities of interest.

Although there exist many bioinformatics graph analysis tools that are freely available, they present at least one of the following limitations. The first limitation we encounter is that many of these recent approaches are limited to the analysis of features in a single homogeneous network, i.e. they consider one network of entities of the same type or domain (e.g. a protein-protein interaction network or a gene network). For instance, *RANKS* [2] performs node prioritization on some label o property by using kernelized score functions, taking into account both the global structure of the network and the neighborhood of the query nodes. Other approaches, like *SVD-phy*, try to find functional associations between genes based on their phylogenetic distributions [3]. Some

approaches, as *DRaWR* [4], widen the features included in a graph by allowing different type of relations between the nodes (i.e. different types of edges).

Other approaches like *FunRich* [5] increase the level of flexibility, allowing users to choose from different data sources to perform enrichment analysis, including the possibility to use a customized database.

On the other hand, there are approaches that allow to include more than one network in the analysis or prioritization task, including different types of interacting entities. However, these methods are built *ad-hoc* to solve a specific problem. Many of these approaches have been proposed for the identification of novel gene-disease potential associations [6], or drug-disease associations for drug repositioning [7]. These methods usually focus more on the data sources integrated into the network than on the algorithm used to propagate the information within and/or accross networks, or they provide an algorithm that is tightly coupled to the data sources in use. In this sense, they lack the possibility of adding new data sources to populate the networks or integrating additional networks with other biomedical entities. Furthermore, the application of these methods to new domains is very challenging, since software and data are tightly coupled.

Since biological analyses can include a wide range of interconnected entities, tools that are able to integrate knowledge from different entity types and sources of data in the form of networks are of interest. Furthermore, the continuous appearance of new data sources to choose from hampers the maintenance of an up-to-date database list.

ProphTools intends to tackle these problems by implementing a general and flexible open-source model for representing heterogeneous networks composed of an arbitrary number of entity types (subnetworks) to perform any user-defined prioritization. ProphTools is based on an approach that has been proven useful in several prioritization applications, such as gene-disease prioritization [8] and drug repositioning [9]. Nonetheless, this functionality has never been made available as

general-purpose software.

In this paper we present ProphTools, an open-source, customizable tool which can be used out-of-the-box for a wide range of prioritization applications. To illustrate this, we applied ProphTools to a prioritization case study on long non-coding RNAs (lncRNAs) and diseases and compared its peformance with recent *ad-hoc* approaches proposed for this task. Further, the data to perform state-of-the-art drug repositioning and lncRNA-disease prioritization using ProphTools have also been made available [10, 11].

2 Methods

2.1 Approach

ProphTools takes as input a heterogeneous network representation file. A heterogeneous network is composed of: i) an arbitrary number of homogeneous subnetworks, each representing biological entities of the same type and their relations; and ii) a set of bipartite subnetworks representing connections between entities of different types. A diagram showing the information included in this file can be seen in figure 1. This representation includes a weighted adjacency matrix for each subnetwork, a bipartite adjacency matrix for each subnetwork-subnetwork relation, and a super-adjacency matrix that provides information about which adjacency matrix correspond to which entity relations. These matrix files can be built using scipy.sparse.io library, which is free and open-source. Node labels are also included in the input file. The specific format is thoroughly explained in ProphTools documentation.

Finally, to complete the definition of the prioritization task, ProphTools requires the user to define one entity network to be the "Query network" and other entity network to be the "Target network".

Given this heterogeneous network configuration, ProphTools can run prioritization queries obtaining the entities from the target network more strongly related to a set of query entities of interest. The proposed representation for the heterogeneous network is general and can fit most of the prioritization tasks proposed in the literature.

ProphTools prioritization methodology works as follows. For a set of nodes in a query network, ProphTools will return a scored list of nodes in the target network, according to their relation with the query dataset. This is performed combining a within-network propagation method similar to Flow Propagation that uses Random Walk with Restarts and a weighted across-network propagation [8]. This two-step process is performed for each path that connects the query network to the target network, and propagation results are eventually compared to results of propagating the nodes on the target network by correlation, a method that has been proven effective in other network-based approaches [12]. In addition, ProphTools can also run Leave One Out with Cross Validation (LOOCV) tests to assess the performance of a given network configuration. For instance, if a drug-target-disease network configuration is provided, ProphTools' ability to predict drug-disease relations can be estimated by performing a 5-fold LOO-CV test on such relation. This predicting capability can be fine-tuned for better performance by testing several network configurations. The results are provided in the form of a ROC curve, an AUC value and a mean rank for each connection removed.

Furthermore, ProphTools provides preprocessing tools to normalize and precompute adjacency values that will be used to speed up the computation time.

2.2 Implementation

ProphTools is implemented in python and does not require high computational resources, although memory requirements may increase with the size and density of the provided networks. The

proposed package is built on broadly used python libraries that are freely available for download, such as numpy high performance array operation library, scipy, and sklearn Machine Learning library [13]. The core propagation method has been sistematically tested using unit testing with a coverage of 80% percent for the entire package. In addition, Travis CI [14] platform for Continuous Integration has been integrated with its repository in order to guarantee its successful deployment on a broad set of computers that meet its reduced software requirements.

Although ProphTools has been developed and tested natively in Linux, it relies on multiplatform libraries. ProphTools is available on GitHub as a python package installable by pip [15]. In order to ensure that ProphTools can run in a wider set of computers, a Docker. version has also been developed. ProphTools Docker version is freely available at DockerHub [16], allowing users of any operating system to easily run ProphTools as long as they have the Docker application installed.

Furthermore, ProphTools is open-source and highly modular, allowing the users to easily extend it with customized within network propagation functions and correlation.

3 Case of study. Long non-coding RNA-disease prioritization.

Recent improvements in sequencing technology have proven that although less than 2% of the human genome codes for genes, more than 85% of the DNA is transcribed [17]. Whereas several types of these non-coding RNAs have been extensively studied, such as micro RNAs and transfer RNAs [18], long non-coding RNAs (lncRNAs) are drawning an increasing interest in the recent years. A recent study estimates in around 58,000 the amount of *loci* transcribing lncRNAs [19]. LncRNAs are, therefore, almost three times as abundant as coding genes according to our current knowledge of the human genome. However, little is known today about these biological entities, although it has been proven that lncRNAs play roles in cell regulation [20] and diseases [21].

Due to the increased relevance that long non-coding RNAs have acquired in the scientific community in the recent years, several *in-silico* and *ad-hoc* approaches have been published to systematically predict new relations between lncRNAs and diseases. LncRNAdisease [22] is a database including experimentally validated relations of lncRNAs and diseases and predictions based on these instances. *LRLSLDA* [22] defines a classification function based on the assumption that similarity between diseases can be an indicator of the similarity between lncRNAs they are associated to. Later, their authors released *IRWRLDA* [23], a network-based lncRNA-disease prioritization algorithm that uses disease semantic similarity and lncRNA expression data to relate lncRNAs, and a modification of a Random Walk with Restarts (RWR) algorithm to perform prioritization. *RWRlncD* [24] also implements RWR on a lncRNA similarity network. LncRNA similarity is also based on the disease sets each lncRNA is associated to in *lncRNAdisease* database [22]. A disadvantage of this method is that it can only perform prioritization on lncRNAs that are associated to at least one disease, which are a very small proportion (156) of the total number of lncRNAs annotated in the human genome (currently 15787 lncRNA annotations in the latest release of GENCODE). More recently, Yao *et al.* proposed *LncPriCNet* [25], a method that built a multi-level network in order to perform lncRNA-disease prioritization.

All these approaches are *ad-hoc* methods developed to solve the lncRNA-disease prioritization problem, not available as general source code. Additionally, the current lack of knowledge about lncRNAs and their relation to disease makes it probably difficult to draw conclusions about a broad set of lncRNAs, since available functional annotations are about two orders of magnitude smaller than the global amount of lncRNA candidates. Due to the interest these biological entities have drawn in the recent years, it seems very likely that this knowledge grows in the near future, and more lncRNA-disease annotations will be made available. However, users will not be able to include future knowledge in these methods, as they are not available as flexible, general-purpose tools.

Here, we apply ProphTools to lncRNA-disease prioritization, as a proof of concept. To do so, we need to model this problem to fit the proposed heterogeneous network representation. Figure 2 shows the network configuration chosen to integrate the available data on lncRNAs and diseases.

Although ProphTools has not been specifically designed to accomplish this particular problem, obtained results are consistent with the current knowledge about lncRNAs and ProphTools is proven as effective as other state-of-the art *ad-hoc* methodologies. Furthermore, the datasets built are freely available to the scientific community to ensure reproducibility and allow further research and improvements on the topic.

3.1 Data

The heterogeneous network configuration includes two entity subnetworks: long non-coding RNAs (lncRNAs) and diseases, and a relation subnetwork lncRNA-disease connecting them (figure 2).

LncRNA network was built using GENCODE v26 [26, 27]. The 15787 lncRNA gene annotations present in GENCODE v26 were processed by generating a projection of overlapping exons for each lncRNA and building a projected transcript representative of each lncRNA. The sequence of each projected transcript was then obtained from the repeat masked version of the human genome hg38. In order to reflect the modular functionality present in lncRNAs [28], we represented each lncRNA gene as a vector of hexamers (short subsequences of 6 nucleotides length). For each lncRNA gene sequence, the appearances of each of the 4096 possible hexamers were counted. These vectors were compared to each other to build an adjacency matrix using as similarity measure the cosine similarity between the hexamer occurrence vectors. These similarities were used as edge weight in our lncRNA network. Additionally, the obtained adjacency matrix was postprocessed removing 50% of the edges, in order to remove propagation noise while keeping the whole network as a single connected element. After this process, 125 nodes (lncRNA genes) were

isolated and removed from the final network, which connects 15662 lncRNAs.

The disease network was obtained from the Disease Ontology, applying the same processing as described for Drugnet [9]. The resulting network includes 4517 diseases that correspond to leaf nodes in the Disease Ontology.

Finally, the lncRNA-disease network was built from lncRNAdisease database [22]. A file corresponding to 1102 experimentally-validated lncRNA-disease connections was downloaded from the lncRNAdisease website [29]. After removing duplicated connections in the lncRNA-disease file, 687 edges were obtained. Naming conventions used in this file for lncRNAs and diseases needed to be matched to GENCODE and the Disease Ontology identifiers, respectively. The ID matching process was performed using fuzzy string matching based on Levenshtein Distance [30] and results were manually revised. The lncRNA id matching process resulted in a set of 229 matches out of 377, after removing general characterizations of sets of lncRNAs (e.g. RNA polymerase III-dependent lncRNAs), and filtering for human long non-coding RNAs. The disease matching process was performed using the same fuzzy string matching library, but two correspondence files were generated: one allowing multiple matches for each lncRNAdisease identifier, and one storing only the best match. Since our disease network includes leaf nodes from the Disease Ontology, non-specific identifiers such as "cancer" or "leukemia" correspond to a set of nodes in our disease network. The multiple matches approach is thus including even more redundancy in the network. To quantify and evaluate this effect in the results, we tested with both options.

After this identifier matching process we obtained two test datasets: i) a `generic` dataset, consisting of 837 edges, including multiple synonyms for generic terms such as "cancer" [see Additional File 1], and ii) a `specific` dataset, consisting of 352 edges where only the best match for the generic terms was included [see Aditional File 2]. Both files can be found in the supplementary material.

3.2 Results

This heterogeneous network configuration was then tested for performance using Leave One Out with 5-fold Cross Validation. This functionality is also implemented in the ProphTools package we provide. Since our disease network includes leaf nodes from the Disease Ontology, non-specific disease associations such as "cancer" or "leukemia" do not yield a single match, but a set of disease nodes. This results in a set of edges representing one correspondence in the original dataset, which could artificially improve the results in a LOO test, since it would allow a certain degree of redundancy that would persist after removing the tested edge. Taking this into account, we have performed two additional versions of the LOOCV test, namely i) a semi-strict version that removes at once all edges connecting a certain entity with the destination network in the direction of propagation, and ii) a strict version that removes all edges connecting the entities in both sides of the test edge (i.e. a semi-strict LOO for both directions of propagation). As figures 3 and 4 show, the AUC value is strongly affected by these two tests, specially if the direction of prioritization is lncRNA-disease (i.e. queries are lncRNAs and targets are diseases). Interestingly, if we perform a disease-lncRNA prioritization (queries are diseases and targets are lncRNAs) the semi-strict and strict LOO tests have lower impact on the final results. This could be related to the Disease Ontology semantic similarity structure, that generates groups of strongly related nodes, such as families of diseases. Furthermore, the lncRNA-network is scarcely populated. The amount of edges provided by the test datasets (837 `general`, 352 `specific`) is very reduced compared to the amount of diseases and lncRNAs in the lncRNA and disease networks, and there are groups of diseases, such as cancer, that cover a high percentage of the total dataset ($\sim$ 23.66 % for the `general` dataset and $\sim$ 18.18 for the `specific` dataset). Performing a strict LOO test can eliminate not only the synonyms introduced in the `general` dataset, but also additional information that comes from a

different source. If many of these cases occur, the resulting prioritization method tries to propagate from one network to another through very few connecting interactions, resulting in poor correlation scores. We believe this effect would be alleviated by a more populated lncRNA-disease network. However, it is interesting to note that although the strict LOO performs poorly for lncRNA-disease prioritization, the semi-strict LOO results are not affected by the removal of synonyms.

Normal, semi-strict and strict LOO results are reported in tables 1 and 2. Normal LOO tests show a 0.963 ± 0.008 AUC value for lncRNA-disease prioritization and a 0.888 ± 0.015 AUC for disease-lncRNA prioritization for the `general` dataset (see table 1), and a 0.850 ± 0.030 AUC value for lncRNA-disease prioritization and a 0.886 ± 0.012 AUC for disease-lncRNA prioritization for the `specific` dataset (see table 2). These results show that predictions made by ProphTools with the proposed heterogeneous network configuration are consistent with current knowledge about lncRNAs and diseases and therefore likely to provide new predictions of interest. These AUC values are competitive with state-of-the art *ad-hoc* approaches, such as *IRWRLDA* [23] (0.7242 and 0.7872 AUC values), *LRLSLDA* [31] (0.7760 AUC value), and *RWRlncD* [24] (0.822 AUC value), and the recent *LncPriCNet* [25] (0.93 AUC value).

4 Conclusions

ProphTools is an open-source, flexible, modular and ready-to-use general implementation of an heterogeneous propagation algorithm that has been proven useful for relevant applications such as gene-disease prioritization and drug repositioning. The abstraction data layer we provide allows users to run ProphTools in any dataset of interest. As a proof of these features, a case study on lncRNA-disease prioritization has been described. Results are competitive with state-of-the art approaches in the field, proving that ProphTools can be of interest to analyse any dataset. In order

**LOO mode**	**propagation direction**	**Mean AUC**		**Mean Rank**		**Mean Rank %**	
normal	lncRNA	0.963	± 0.008	522.16	± 120.21	3.33	± 0.77
	disease	0.888	± 0.015	503.67	± 69.57	11.15	± 1.54
semi-strict	lncRNA	0.917	± 0.008	1240.95	± 130.76	7.92	± 0.83
	disease	0.854	± 0.018	655.17	± 78.45	14.50	± 1.74
strict	lncRNA	0.823	± 0.016	2767.30	± 241.71	17.67	± 0.01
	disease	0.618	± 0.096	1725.18	± 435.05	38.19	± 9.63

Table 1: **ProphTools performance results on the general lncRNA-disease dataset for three different 5-fold LOO-CV modes**. Normal LOO removes only one edge per test. Semi-strict LOO mode removes all edges including origin node towards propagation direction, and strict mode removes all edges involving the two nodes connected by each test edge. Propagation direction shows whether lncRNAs or diseases are are being ranked. Mean AUC column shows the average AUC obtained at the 5-fold LOO test for each category. Mean Rank shows the average ranking obtained for each test case, and Mean Rank% shows the rank obtained in terms of percentage.

**LOO mode**	**propagation direction**	**Mean AUC**		**Mean Rank**		**Mean Rank %**	
normal	lncRNA	0.886	± 0.012	1751.24	± 194.68	11.18	± 1.24
	disease	0.850	± 0.030	670.48	± 135.53	14.84	± 3.00
semi-strict	lncRNA	0.866	± 0.026	2059.37	± 403.71	13.15	± 2.58
	disease	0.877	± 0.020	548.05	± 91.76	12.13	± 2.03
strict	lncRNA	0.828	± 0.034	2690.47	± 476.54	17.18	± 3.04
	disease	0.602	± 0.058	1794.22	± 261.58	39.72	± 5.79

Table 2: **ProphTools performance results on the specific lncRNA-disease dataset for three different 5-fold LOO-CV modes**. Normal LOO removes only one edge per test. Semi-strict LOO mode removes all edges including origin node towards propagation direction, and strict mode removes all edges involving the two nodes connected by each test edge. Propagation direction shows whether lncRNAs or diseases are are being ranked. Mean AUC column shows the average AUC obtained at the 5-fold LOO test for each category. Mean Rank shows the average ranking obtained for each test case, and Mean Rank% shows the rank obtained in terms of percentage.

to ensure the reproducibility of the results and allow further improvements in lncRNA-disease prioritization, the datasets built to apply ProphTools have also been made available.

ProphTools source code is available both as a GitHub repository and as a standalone python package that can be easily installed via pip [15].

Furthermore, unit testing and continuous integration techniques have been applied during the development of the package in order to guarantee code stability and deployment on a broad set of computers. ProphTools has also been made available as a Docker container [16], making it possible to run it in any platform that has Docker installed. Additionally, ProphTools is not only open-source but also very modular in design, allowing advanced users to extend its functionality. We are already working on further features (such as additional propagation algorithms) to incorporate to the ProphTools framework in future versions.

Finally, we expect that the availability of our prioritization method as an open-source, customizable tool can be of use for a wide range of biological applications.

5 Availability of supporting source code and requirements

Project name: ProphTools.

Project home page: `https://github.com/cnluzon/prophtools`, `https://hub.docker.com/r/cnluzon/prophtools/`

Operating system(s): Linux, other operating systems if using the Docker version.

Programming language: Python 2.7

Other requirements: Non-linux systems need to run the Docker version. Native linux systems require following python libraries (installed automatically when installing via pip): numpy (>=1.11.2), scipy (>=0.18.1), matplotlib (>=1.4.3), scikit-learn (>=0.18), networkx (>=1.11).

License: GNU GPLv3.0.

6 Availability of data and materials

ProphTools source code available at GitHub [15] and as a Docker container at Docker hub [16]. Heterogeneous network configurations for lncRNA-disease prioritization are available for download at ProphTools website [11]. Drug-gene-disease prioritization data is also available at our server [10].

7 Declarations

List of abbreviations

- lncRNA. Long non-coding RNA.
- RWR. Random Walk with Restarts.
- LOO. Leave One Out.
- LOOCV. Leave One Out with Cross-Validation.

Ethics approval and consent to participate

Not applicable.

Consent for publication

Not applicable

Competing interests

The authors declare that they have no competing interests.

Funding

This work was supported by Junta de Andalucía [PI-0710-2013]; Dirección General de Investigación Científica y Técnica [TIN2013-41990-R]; European Regional Development Fund; and Spanish Ministry of Education, Culture and Sports [C. Navarro's FPU grant].

Authors' contributions

CN developed the software, both the python repository and the Dockerized version, performed data analysis and wrote the paper. VM developed the methodology and participated in the core software functionality. CC supervised the development of the software and data analysis, reviewed and edited the paper. AB conceptualized the research idea, supervised the quality of the process and also reviewed and edited the draft.

Acknowledgements

Not applicable.

References

[1] Jessica Xin Hu, Cecilia Engel Thomas, and Søren Brunak. Network biology concepts in complex disease comorbidities. *Nature Reviews Genetics*, 2016.

[2] Giorgio Valentini, Giuliano Armano, Marco Frasca, Jianyi Lin, Marco Mesiti, and Matteo Re. Ranks: a flexible tool for node label ranking and classification in biological networks. *Bioinformatics*, page btw235, 2016.

[3] Andrea Franceschini, Jianyi Lin, Christian von Mering, and Lars Juhl Jensen. Svd-phy: improved prediction of protein functional associations through singular value decomposition of phylogenetic profiles. *Bioinformatics*, page btv696, 2015.

[4] Charles Blatti and Saurabh Sinha. Characterizing gene sets using discriminative random walks with restart on heterogeneous biological networks. *Bioinformatics*, page btw151, 2016.

[5] Mohashin Pathan, Shivakumar Keerthikumar, Ching-Seng Ang, Lahiru Gangoda, Camelia YJ Quek, Nicholas A Williamson, Dmitri Mouradov, Oliver M Sieber, Richard J Simpson, Agus Salim, et al. Funrich: An open access standalone functional enrichment and interaction network analysis tool. *Proteomics*, 15(15):2597–2601, 2015.

[6] Yves Moreau and Léon-Charles Tranchevent. Computational tools for prioritizing candidate genes: boosting disease gene discovery. *Nature Reviews Genetics*, 13(8):523–536, 2012.

[7] Zerrin Isik, Christoph Baldow, Carlo Vittorio Cannistraci, and Michael Schroeder. Drug target prioritization by perturbed gene expression and network information. *Scientific reports*, 5, 2015.

[8] Víctor Martínez, Carlos Cano, and Armando Blanco. Prophnet: A generic prioritization method through propagation of information. *BMC bioinformatics*, 15(1):1, 2014.

[9] Víctor Martínez, Carmen Navarro, Carlos Cano, Waldo Fajardo, and Armando Blanco. Drugnet: Network-based drug–disease prioritization by integrating heterogeneous data. *Artificial intelligence in medicine*, 63(1):41–49, 2015.

[10] Carmen Navarro, Víctor Martínez, Carlos Cano, and Armando Blanco. Drug-gene-disease prioritization prioritization heterogeneous network configuration supplementary material. `http://genome.ugr.es:9000/download/data/drugnet_data_non_precomputed.zip`. Accessed May 26, 2017.

[11] Carmen Navarro, Víctor Martínez, Carlos Cano, and Armando Blanco. Lncrna-disease prioritization heterogeneous network configuration supplementary material. `http://genome.ugr.es:9000/download/data/lncrna_disease_prophtools_general.mat`. Accessed May 26, 2017.

[12] TaeHyun Hwang, Wei Zhang, Maoqiang Xie, Jinfeng Liu, and Rui Kuang. Inferring disease and gene set associations with rank coherence in networks. *Bioinformatics*, 27(19):2692–2699, 2011.

[13] Fabian Pedregosa, Gaël Varoquaux, Alexandre Gramfort, Vincent Michel, Bertrand Thirion, Olivier Grisel, Mathieu Blondel, Peter Prettenhofer, Ron Weiss, Vincent Dubourg, et al. Scikit-learn: Machine learning in python. *Journal of Machine Learning Research*, 12(Oct):2825–2830, 2011.

[14] Travis ci continuous integration platform. `https://travis-ci.org/`. Accessed May 26, 2017.

[15] Carmen Navarro, Victor Martínez, Carlos Cano, and Armando Blanco. Prophtools: General prioritization tools for heterogeneous biological networks., 2017. GitHub repository. `http://www.github.com/cnluzon/prophtools`. Accessed May 26, 2017.

[16] Carmen Navarro, Víctor Martínez, Carlos Cano, and Armando Blanco. Prophtools: General prioritization tools for heterogeneous biological networks. Docker Container. `https://hub.docker.com/r/cnluzon/prophtools/`. Accessed May 26, 2017.

[17] Matthew J Hangauer, Ian W Vaughn, and Michael T McManus. Pervasive transcription of the human genome produces thousands of previously unidentified long intergenic noncoding rnas. *PLoS Genet*, 9(6):e1003569, 2013.

[18] Ewan A Gibb, Carolyn J Brown, and Wan L Lam. The functional role of long non-coding rna in human carcinomas. *Molecular cancer*, 10(1):1, 2011.

[19] Matthew K Iyer, Yashar S Niknafs, Rohit Malik, Udit Singhal, Anirban Sahu, Yasuyuki Hosono, Terrence R Barrette, John R Prensner, Joseph R Evans, Shuang Zhao, et al. The landscape of long noncoding rnas in the human transcriptome. *Nature genetics*, 47(3):199–208, 2015.

[20] Chris P Ponting, Peter L Oliver, and Wolf Reik. Evolution and functions of long noncoding rnas. *Cell*, 136(4):629–641, 2009.

[21] Orly Wapinski and Howard Y Chang. Long noncoding rnas and human disease. *Trends in cell biology*, 21(6):354–361, 2011.

[22] Geng Chen, Ziyun Wang, Dongqing Wang, Chengxiang Qiu, Mingxi Liu, Xing Chen, Qipeng Zhang, Guiying Yan, and Qinghua Cui. Lncrnadisease: a database for long-non-coding rna-associated diseases. *Nucleic acids research*, 41(D1):D983–D986, 2013.

[23] Xing Chen, Zhu-Hong You, Gui-Ying Yan, and Dun-Wei Gong. Irwrlda: improved random walk with restart for lncrna-disease association prediction. *Oncotarget*, 7(36):57919, 2016.

[24] Jie Sun, Hongbo Shi, Zhenzhen Wang, Changjian Zhang, Lin Liu, Letian Wang, Weiwei He, Dapeng Hao, Shulin Liu, and Meng Zhou. Inferring novel lncrna–disease associations based on a random walk model of a lncrna functional similarity network. *Molecular BioSystems*, 10(8):2074–2081, 2014.

[25] Qianlan Yao, Leilei Wu, Jia Li, Li guang Yang, Yidi Sun, Zhen Li, Sheng He, Fangyoumin Feng, Hong Li, and Yixue Li. Global prioritizing disease candidate lncrnas via a multi-level composite network. *Scientific Reports*, 7, 2017.

[26] Thomas Derrien, Rory Johnson, Giovanni Bussotti, Andrea Tanzer, Sarah Djebali, Hagen Tilgner, Gregory Guernec, David Martin, Angelika Merkel, David G Knowles, et al. The gencode v7 catalog of human long noncoding rnas: analysis of their gene structure, evolution, and expression. *Genome research*, 22(9):1775–1789, 2012.

[27] Gencode project homepage. `http://gencodegenes.org`. Accessed May 26, 2017.

[28] Tim R Mercer and John S Mattick. Structure and function of long noncoding rnas in epigenetic regulation. *Nature structural & molecular biology*, 20(3):300–307, 2013.

[29] Geng Chen, Ziyun Wang, Dongqing Wang, Chengxiang Qiu, Mingxi Liu, Xing Chen, Qipeng Zhang, Guiying Yan, and Qinghua Cui. Lncrnadisease: a database for long-non-coding rna-associated diseases (website). `http://www.cuilab.cn/lncrnadisease`. Accessed May 26, 2017.

[30] Fuzzy wuzzy fuzzy string matching python library. `https://github.com/seatgeek/fuzzywuzzy`. Accessed May 26, 2017.

[31] Xing Chen and Gui-Ying Yan. Novel human lncrna–disease association inference based on lncrna expression profiles. *Bioinformatics*, page btt426, 2013.

Figures

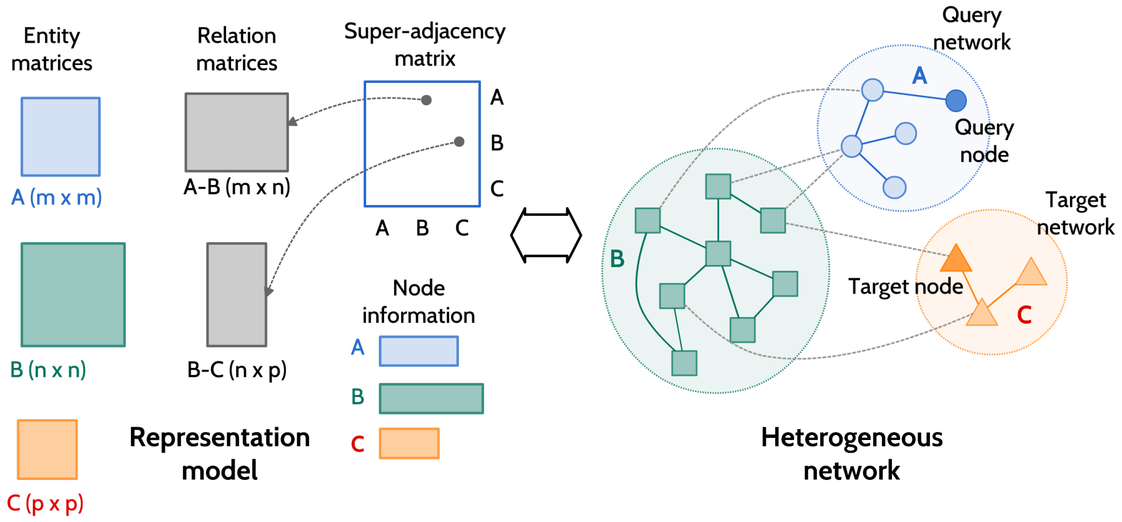

Figure 1: ProphTools heterogeneous network representation model. For each subnetwork, an adjacency matrix is provided. Additionally, connections between different entities are modelled as bipartite adjacency matrices. Finally, a super-adjacency matrix models how each relation matrix connects two entity matrices. Node labels are also stored in this representation.

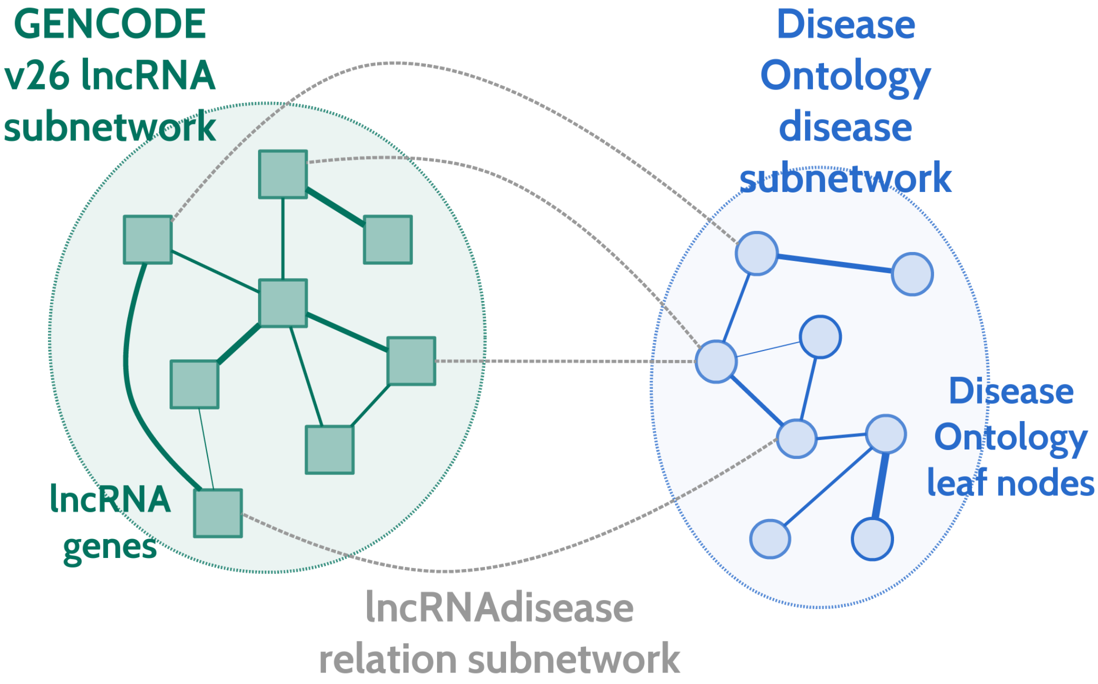

Figure 2: Heterogeneous network configuration built to perform lncRNA-disease ProphTools prioritization. LncRNA subnetwork is built from GENCODE v26 lncRNA sequences. Disease subnetwork is built from Disease Ontology leaf nodes using semantic similarity measures as in DrugNet [9]. LncRNA-disease relation subnetwork is taken from lncRNAdisease database [22]. This data configuration file is available at ProphTools website.

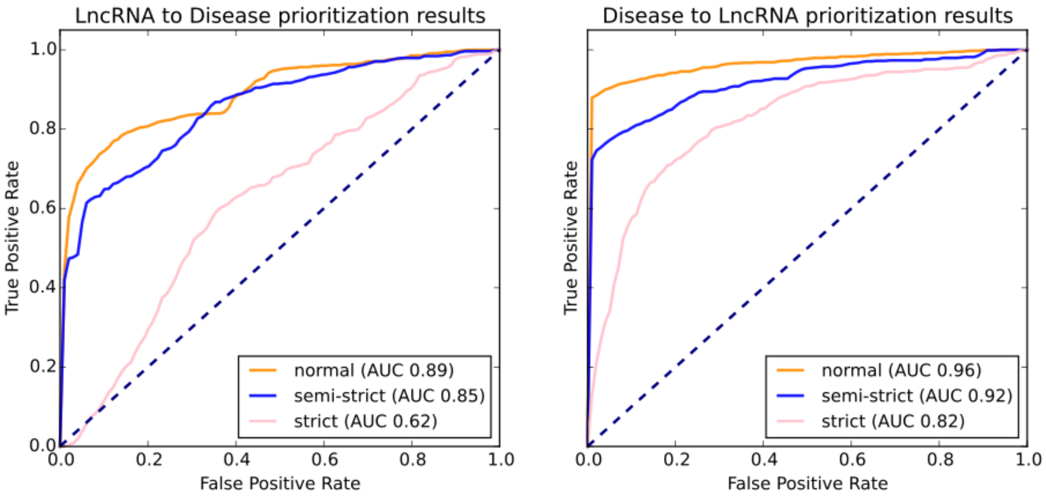

Figure 3: Results for 5-fold LOO-CV tests on the `general` dataset obtained from experimental evidence available in LncRNAdisease database. On the left side, ROC curves obtained for the normal, semi-strict and strict LOO performed from lncRNA to disease. On the right side, ROC curves obtained from for disease to lncRNA prioritization.

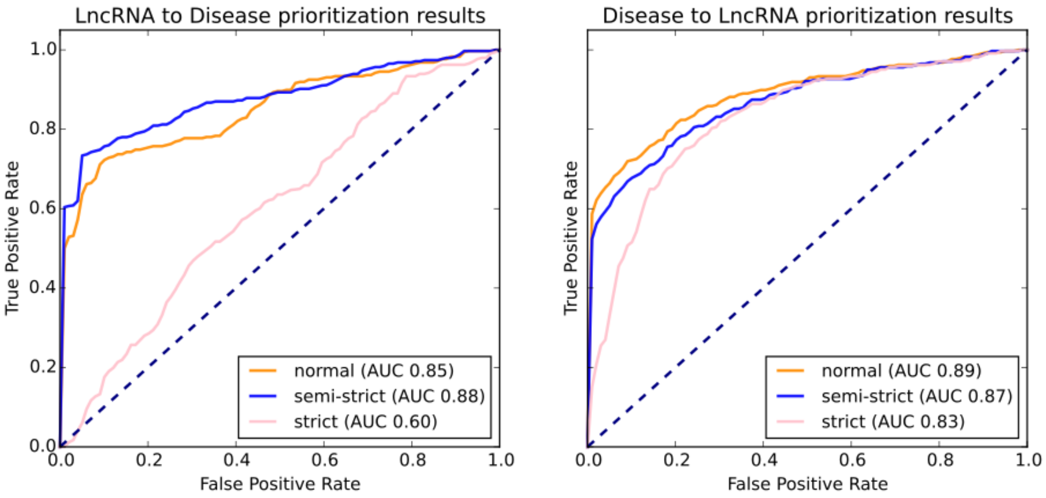

Figure 4: Results for 5-fold LOO-CV tests on the `specific` dataset obtained from experimental evidence available in LncRNAdisease database. On the left side, ROC curves obtained for the normal, semi-strict and strict LOO performed from lncRNA to disease. On the right side, ROC curves obtained from for disease to lncRNA prioritization.

Figure 1

Click here to download Figure fig01_matfile.png

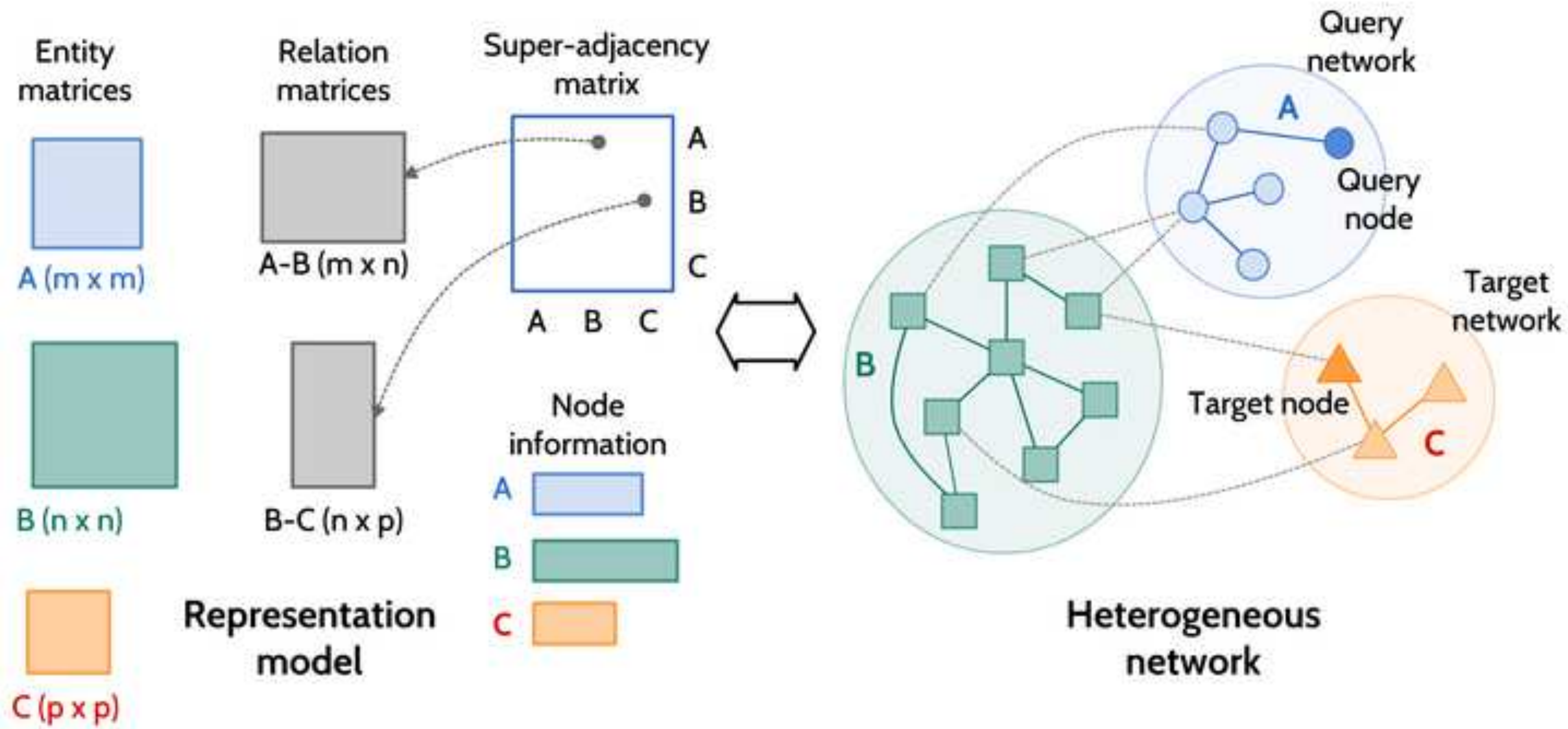

Figure 2

Click here to download Figure fig02_lncrnadisease.png

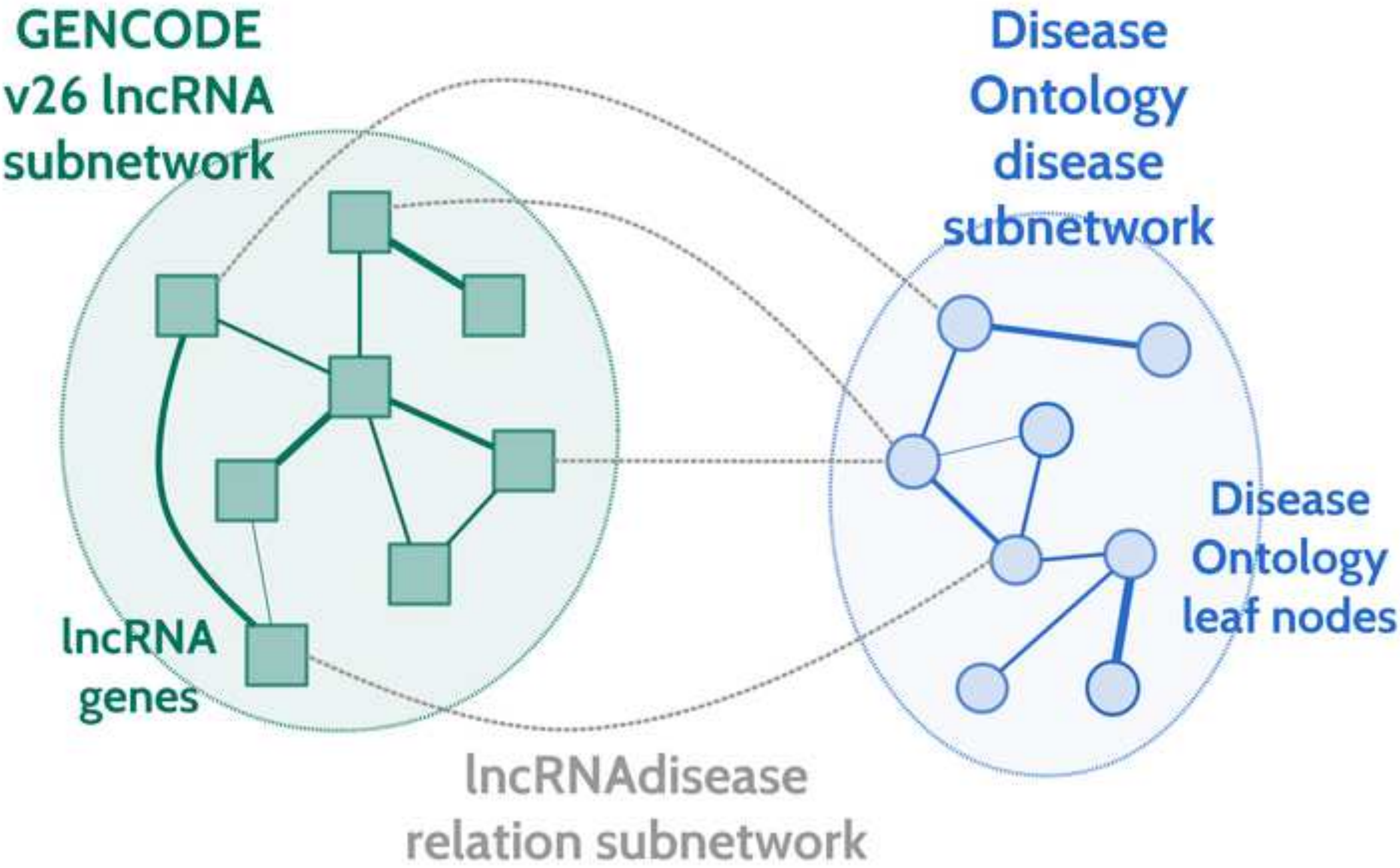

Figure 3 Click here to download Figure fig03_lootests.png

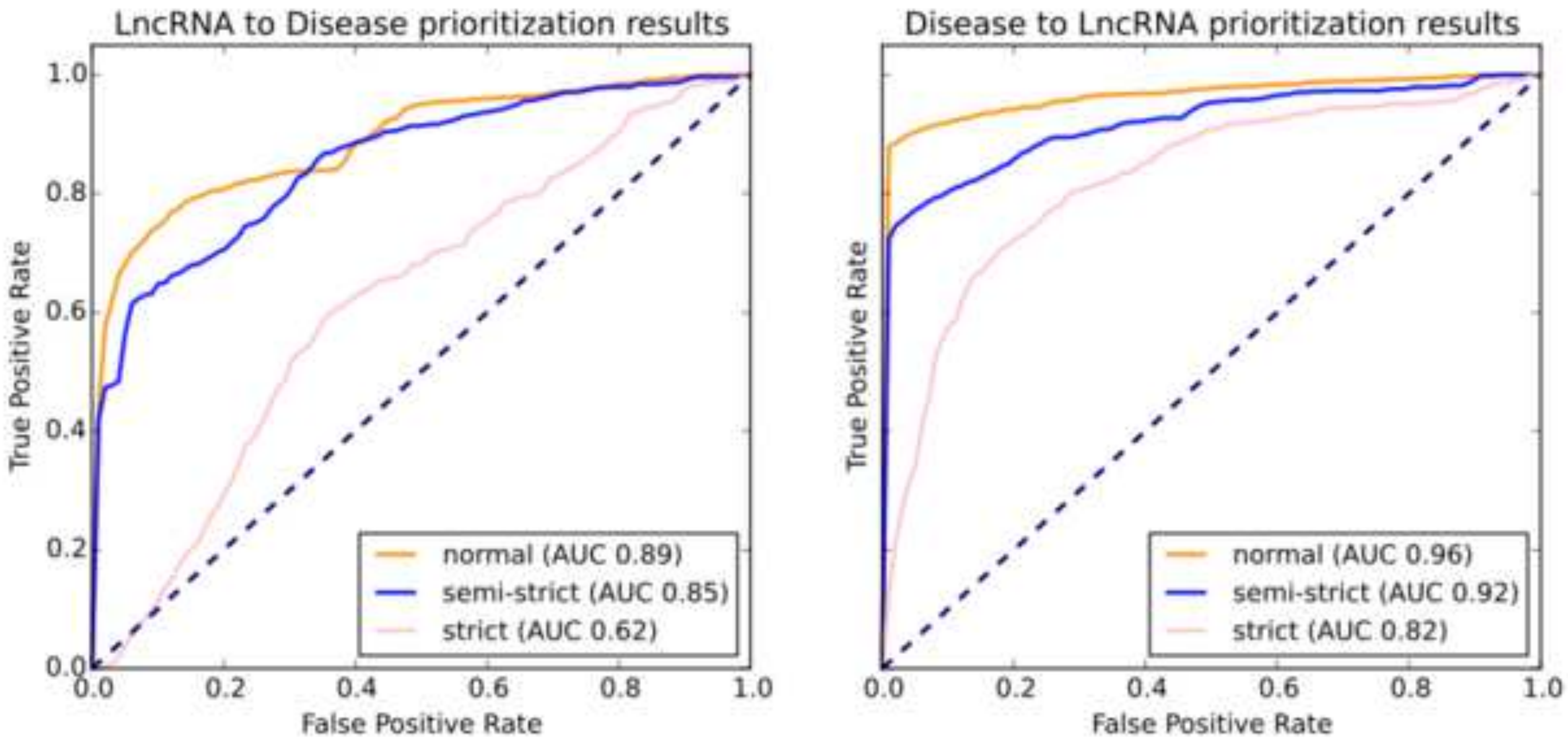

Figure 4

Click here to download Figure fig04_lootestsspecific.png

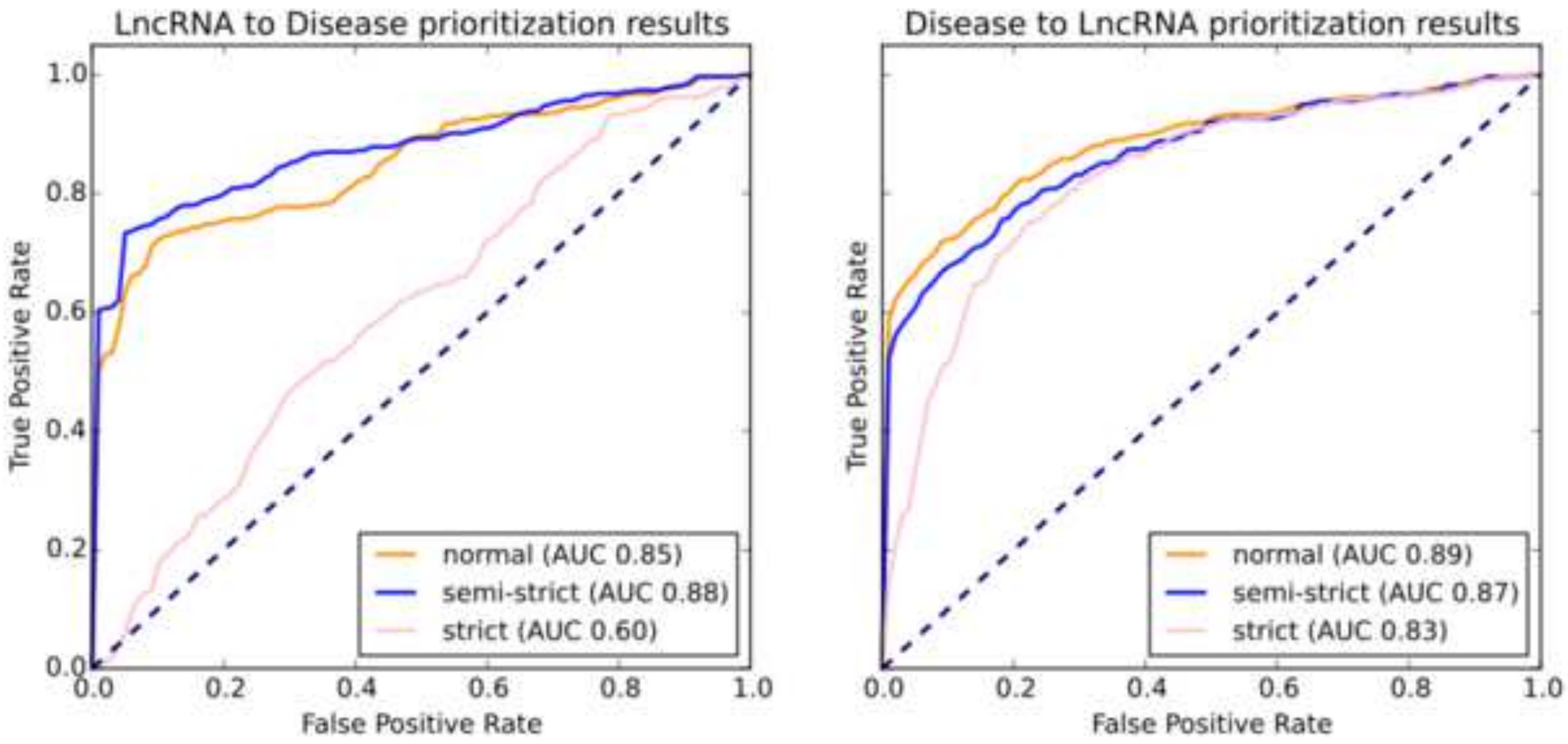

Additional File 1. General dataset.

Click here to access/download
Supplementary Material
SupFile01_lncrna_disease_prophtools_general.csv

Additional File 1 description file.

Click here to access/download
Supplementary Material
SupFile01_lncrna_disease_prophtools_general_info.txt

Additional File 2. Specific dataset.

Click here to access/download

Supplementary Material

SupFile02_lncrna_disease_prophtools_specific.csv

Additional File 2 description file.

Click here to access/download
Supplementary Material
SupFile02_lncrna_disease_prophtools_specific_info.txt
