# Supplementary material for: ProphTools: general prioritization tools for heterogeneous biological networks
Source: Gigascience. 2017 Nov 24;6(12):1–8. doi: 10.1093/gigascience/gix111 (PMC5751048; doi:10.1093/gigascience/gix111)
Supplement: GIGA-D-17-00123_Revision-2.pdf [file gix111_giga-d-17-00123_revision-2.pdf]

GigaScience

ProphTools: General Prioritization Tools for Heterogeneous Biological Networks

--Manuscript Draft--

**Manuscript Number:**	GIGA-D-17-00123R2
**Full Title:**	ProphTools: General Prioritization Tools for Heterogeneous Biological Networks
**Article Type:**	Technical Note
**Funding Information:**	Dirección General de Investigación Científica y Técnica (TIN2013-41990-R) — Dr. Armando Blanco Morón<br>European Regional Development Fund — Dr. Armando Blanco Morón<br>Ministerio de Educación, Cultura y Deporte (FPU12_01875) — Ms. Carmen Navarro Luzón<br>Dirección General de Investigación Científica y Técnica (DPI2017-84439-R) — Dr. Armando Blanco Morón
**Abstract:**	Background: Networks have been proven effective representations for the analysis of biological data. As such, there exist multiple methods to extract knowledge from biological networks. However, these approaches usually limit their scope to a single biological entity type of interest, or they lack flexibility to analyse user-defined data.<br>Results: We developed ProphTools, a flexible open-source command-line tool that performs prioritization on a heterogeneous network.<br>ProphTools prioritization combines a Flow Propagation algorithm similar to a Random Walk With Restarts and a weighted propagation method. A flexible model for the representation of a heterogeneous network allows the user to define a prioritization problem involving an arbitrary number of entity types and their interconnections. Furthermore, ProphTools provides functionality to perform Cross-Validation tests, allowing users to select the best network configuration for a given problem. ProphTools core prioritization methodology has already been proven effective in gene-disease prioritization and drug repositioning. Here we make ProphTools available to the scientific community as flexible, open-source software and perform a new proof-of-concept case study on long non-coding RNAs (lncRNAs) to disease prioritization.<br>Conclusions: ProphTools is robust prioritization software that provides the flexibility not present in other state-of-the-art network analysis approaches, enabling researchers to perform prioritization tasks on any user-defined heterogeneous network. Furthermore, the application to lncRNA-disease prioritization showed that ProphTools can reach the performance levels of ad-hoc prioritization tools without losing its generality.
**Corresponding Author:**	Carmen Navarro Luzón<br>Universidad de Granada<br>SPAIN
**Corresponding Author Secondary Information:**	
**Corresponding Author's Institution:**	Universidad de Granada
**Corresponding Author's Secondary Institution:**	
**First Author:**	Carmen Navarro Luzón
**First Author Secondary Information:**	
**Order of Authors:**	Carmen Navarro Luzón<br>Víctor Martínez Gómez<br>Armando Blanco Morón<br>Carlos Cano Gutiérrez

Powered by Editorial Manager® and ProduXion Manager® from Aries Systems Corporation

**Order of Authors Secondary Information:**	
**Response to Reviewers:**	Dear editor,<br><br>please find the revised version of the manuscript in this submission. The supporting GigaDB dataset has been added to the references and mentioned in the "Availability of supporting data" section.<br><br>Source latex files are now attached and have been revised for possible typos.<br><br>The proposed tool has also been registerd at SciCrunch and the received ID displayed at "Availability of supporting source code and requirements" section.<br><br>Thank you very much for your comments and suggestions.
**Additional Information:**	
**Question**	**Response**
Are you submitting this manuscript to a special series or article collection?	No
**Experimental design and statistics**<br><br>Full details of the experimental design and statistical methods used should be given in the Methods section, as detailed in our Minimum Standards Reporting Checklist. Information essential to interpreting the data presented should be made available in the figure legends.<br><br>Have you included all the information requested in your manuscript?	Yes
**Resources**<br><br>A description of all resources used, including antibodies, cell lines, animals and software tools, with enough information to allow them to be uniquely identified, should be included in the Methods section. Authors are strongly encouraged to cite Research Resource Identifiers (RRIDs) for antibodies, model organisms and tools, where possible.<br><br>Have you included the information requested as detailed in our Minimum Standards Reporting Checklist?	Yes
**Availability of data and materials**<br><br>All datasets and code on which the conclusions of the paper rely must be either included in your submission or deposited in publicly available repositories (where available and ethically	Yes

appropriate), referencing such data using a unique identifier in the references and in the “Availability of Data and Materials” section of your manuscript.<br><br>Have you have met the above requirement as detailed in our Minimum Standards Reporting Checklist?	

Manuscript

Click here to download Manuscript prophtools_final_version.pdf

OXFORD

(GIGA)^n SCIENCE

GigaScience, 2017, 1–8

doi: xx.xxxx/xxxx
Manuscript in Preparation
Technical note

TECHNICAL NOTE

ProphTools: General Prioritization Tools for Heterogeneous Biological Networks

Carmen Navarro[1,*], Victor Martínez[1], Armando Blanco[1] and Carlos Cano[1]

[1]Department of Computer Science and Artificial Intelligence, University of Granada, Spain

Corresponding email: *cnluzon@decsai.ugr.es

Abstract

Background: Networks have been proven effective representations for the analysis of biological data. As such, there exist multiple methods to extract knowledge from biological networks. However, these approaches usually limit their scope to a single biological entity type of interest, or they lack flexibility to analyse user-defined data.
Results: We developed ProphTools, a flexible open-source command-line tool that performs prioritization on a heterogeneous network. ProphTools prioritization combines a Flow Propagation algorithm similar to a Random Walk With Restarts and a weighted propagation method. A flexible model for the representation of a heterogeneous network allows the user to define a prioritization problem involving an arbitrary number of entity types and their interconnections. Furthermore, ProphTools provides functionality to perform Cross-Validation tests, allowing users to select the best network configuration for a given problem. ProphTools core prioritization methodology has already been proven effective in gene-disease prioritization and drug repositioning. Here we make ProphTools available to the scientific community as flexible, open-source software and perform a new proof-of-concept case study on long non-coding RNAs (lncRNAs) to disease prioritization.
Conclusions: ProphTools is robust prioritization software that provides the flexibility not present in other state-of-the-art network analysis approaches, enabling researchers to perform prioritization tasks on any user-defined heterogeneous network. Furthermore, the application to lncRNA-disease prioritization showed that ProphTools can reach the performance levels of *ad-hoc* prioritization tools without losing its generality.

Key words: Network analysis; prioritization; heterogeneous networks; long non-coding RNAs

Findings

Background

Biological processes are complex and usually involve a large amount of entities interacting with each other. In this sense, it has been proven that networks are an effective model to improve our understanding of such processes, and many methodologies that use a network representation to infer new hypotheses from existing biological knowledge have been made available in the recent years [1]. These approaches model biological entities as nodes in a graph, where weighted edges correspond to interactions or any type of relationship between the connected nodes or entities. Edge weight, in this sense, measures the strength of the represented relationship. Many approaches have been proposed to build biological networks from data sources and to perform inference tasks on them [2].

From protein-protein interaction prediction [3] to the identification of candidate disease genes to drug repositioning [4] or very recent applications on microbiology [5], it seems to be clear that inference on graph or network data structures can be effective for the purpose of finding relations between entities that interact in such ways [1]. These *in-silico* predictions allow researchers to reduce the search space to focus on a small set of entities that are more likely to be related to the entities of interest.

Although there exist many bioinformatics graph analysis tools that are freely available, they present at least one of the

Compiled on: November 8, 2017.
Draft manuscript prepared by the author.

following limitations.

The first limitation we encounter is that many of these recent approaches are limited to the analysis of features in a single homogeneous network, i.e. they consider one network of entities of the same type or domain (e.g. a protein-protein interaction network or a gene network). For instance, *RANKS* [6] performs node prioritization on some label or property by using kernelized score functions, taking into account both the global structure of the network and the neighborhood of the query nodes. Other approaches, like *SVD-phy*, try to find functional associations between genes based on their phylogenetic distributions [7]. Some approaches, as *DRaWR* [8], widen the features included in a graph by allowing different type of relations between the nodes (i.e. different types of edges).

Other approaches like *FunRich* [9] increase the level of flexibility, allowing users to choose from different data sources to perform enrichment analysis, including the possibility to use a customized database.

On the other hand, there are approaches that allow to include more than one network in the analysis or prioritization task, including different types of interacting entities. However, these methods are built *ad-hoc* to solve a specific problem. Many of these approaches have been proposed for the identification of novel gene-disease potential associations [10], or drug-disease associations for drug repositioning [11]. These methods usually focus more on the data sources integrated into the network than on the algorithm used to propagate the information within and/or accross networks, or they provide an algorithm that is tightly coupled to the data sources in use. In this sense, they lack the possibility of adding new data sources to populate the networks or integrating additional networks with other biomedical entities. Furthermore, the application of these methods to new domains is very challenging, since software and data are tightly coupled.

Since biological analyses can include a wide range of interconnected entities, tools that are able to integrate knowledge from different entity types and sources of data in the form of networks are of interest. Furthermore, the continuous appearance of new data sources to choose from hampers the maintenance of an up-to-date database list.

ProphTools intends to tackle these problems by implementing a general and flexible open-source model for representing heterogeneous networks composed of an arbitrary number of entity types (subnetworks) to perform any user-defined prioritization. ProphTools is based on an approach that has been proven useful in several prioritization applications, such as gene-disease prioritization [12] and drug repositioning [13]. Nonetheless, this functionality has never been made available as general-purpose software.

In this paper we present ProphTools, an open-source, customizable tool which can be used for a wide range of prioritization applications. To illustrate this, we applied ProphTools to a prioritization case study on long non-coding RNAs (lncRNAs) and diseases and compared its peformance with recent *ad-hoc* approaches proposed for this task. Further, the data to perform state-of-the-art drug repositioning and lncRNA-disease prioritization using ProphTools have also been made available [14, 15].

Approach

ProphTools methodology operates on a heterogeneous global graph, $G = (D, R)$, where D is a set of entity subnetworks (nodes of the same biological type) and R is a set of relationship subnetworks (bipartite networks connecting two different types of nodes). Given a set of nodes Q from the query network D_q and a target network D_t, the goal prioritization task is to determine the degree of relationship of the nodes in D_t to the query nodes in Q.

ProphTools performs this prioritization combining i) a within-network propagation method similar to Flow Propagation that uses Random Walk with Restarts and ii) a weighted across-network propagation [12]. As Algorithm 1 shows, these processes are repeatedly applied to each network in every path from the query network to the target network. Values propagated from the query network eventually reach the target network and are then compared to values propagated from the target nodes by correlation [16].

Algorithm 1 Prioritization from query subnet D_q to target subnet D_t. G is the global graph and Q the query set.

function <propagate>(G, Q, D_q, D_t)
  Propagate *within-network* in D_q
  P: list of paths from D_q to D_t in G
  for p_i in P **do**
    for subnet p_{ij} in the path p_i from p_{i1} to $p_{i(l-1)}$ **do**
      Propagate values from p_{ij} to $p_{i(j+1)}$
      Propagate values within $p_{i(j+1)}$
    end for
    Store values in $p_{i(l-1)}$ after propagation in p_i as $x_{i(l-1)}$
  end for
  for $e \in V_t$ in target subnetwork D_t **do**
    Set a target set $T = e$
    Propagate values within D_t
    Compute correlation coef. s_e using $x_{i(l-1)}$ for each p_i
  end for
  L: Sort entities in $e \in V$ by values s_e in descending order
  Return L
end function

In addition, ProphTools can also run Cross Validation (CV) tests to assess the performance of a given network configuration. For instance, a 5-fold CV test on such network configuration would remove one fifth of the interactions connecting D_q and D_t and evaluate their predictability from the remaining network structure. The results are provided in the form of a ROC curve, an AUC value and a mean rank for each connection removed. These values can be used to compare the performance of different network configurations.

Implementation

ProphTools is implemented in python and does not require high computational resources, although memory requirements may increase with the size and density of the provided networks. The proposed package is built on broadly used python libraries that are freely available for download, such as numpy high performance array operation library, scipy, and sklearn Machine Learning library [17]. The core propagation method has been sistematically tested using unit testing with a coverage of 86% percent for the entire package. In addition, Travis CI [18] platform for Continuous Integration has been connected with its repository in order to guarantee its successful deployment on a broad set of computers that meet its reduced software requirements.

Although ProphTools has been developed and tested natively in Linux, it relies on multiplatform libraries. ProphTools is available on GitHub as a python package installable by pip [19]. In order to ensure that ProphTools can run in a wider set of computers, a Docker version has also been developed. ProphTools Docker version is freely available at DockerHub [20], allowing users of any operating system to easily run ProphTools

as long as they have the Docker application installed.

Furthermore, ProphTools is open-source and highly modular, allowing users to easily extend it with alternative propagation methods and scoring functions.

How to use

ProphTools uses internally a heterogeneous network representation file. A heterogeneous network is composed of: i) an arbitrary number of homogeneous subnetworks, each representing biological entities of the same type and their relations; and ii) a set of bipartite subnetworks representing connections between entities of different types. A diagram showing the information included in this file can be seen in figure 1. This representation includes a weighted adjacency matrix for each subnetwork, a bipartite adjacency matrix for each subnetwork-subnetwork relation, and a super-adjacency matrix that provides information about which adjacency matrix correspond to which entity relations. These matrix files can be built using scipy.sparse.io library, which is free and open-source. Node labels are also included in the input file. This specific format is thoroughly explained in ProphTools documentation.

This internal network specification allows ProphTools to perform prioritization tasks in any user-specified dataset. Nonetheless, in order to facilitate its application, ProphTools also supports two general network specification formats: a plain text format, and Graph Exchange XML File (GEXF) file format [21].

The plain text format consists of a list of nodes and a list of edges, as in any regular graph, plus a label per node to specify which group each node belongs to. Figure 1 shows an example for a user specified text file with three different subnetworks and its correspondence with ProphTools internal heterogeneous network model.

Furthermore, GEXF file format is based on XML, which is broadly used and flexible, allowing advanced users to ensure compatibility with other tools, such as Gephi graph visualization tool [22].

ProphTools allows users to perform two operations: prioritization (run queries on specific sets of nodes for any of the networks) and performance tests (Cross-Validation).

For example, to apply ProphTools to the network configuration in figure 1, the user would first generate ProphTools internal network model file:

```
prophtools buildmat --file example.txt --format txt
--out example.mat
```

The user can perform any prioritization queries on the resulting `mat` file. For instance, to prioritize target nodes in subnetwork C from the query set $Q = \{0, 1\}$ in subnetwork A:

```
prophtools prioritize --matfile example.mat --src A
--dst C --qname 0,1 --out results.csv
```

To test the global performance of prioritizing target subnetwork C from query subnetwork A, the user could perform a 2-fold CV test:

```
prophtools cross --matfile example.mat --src A --dst C
--fold 2 --out cvresults
```

Additional details and file examples are provided in ProphTools Git repository and documentation.

Case study. Long non-coding RNA-disease prioritization.

Recent improvements in sequencing technology have proven that although less than 2% of the human genome codes for genes, more than 85% of the DNA is transcribed [23]. Whereas several types of these non-coding RNAs have been extensively studied, such as micro RNAs and transfer RNAs [24], long non-coding RNAs (lncRNAs) are drawning an increasing interest in the recent years. A recent study estimates in around 58,000 the amount of *loci* transcribing lncRNAs [25]. LncRNAs are, therefore, almost three times as abundant as coding genes according to our current knowledge of the human genome. However, little is known today about these biological entities, although it has been proven that lncRNAs play roles in cell regulation [26] and diseases [27].

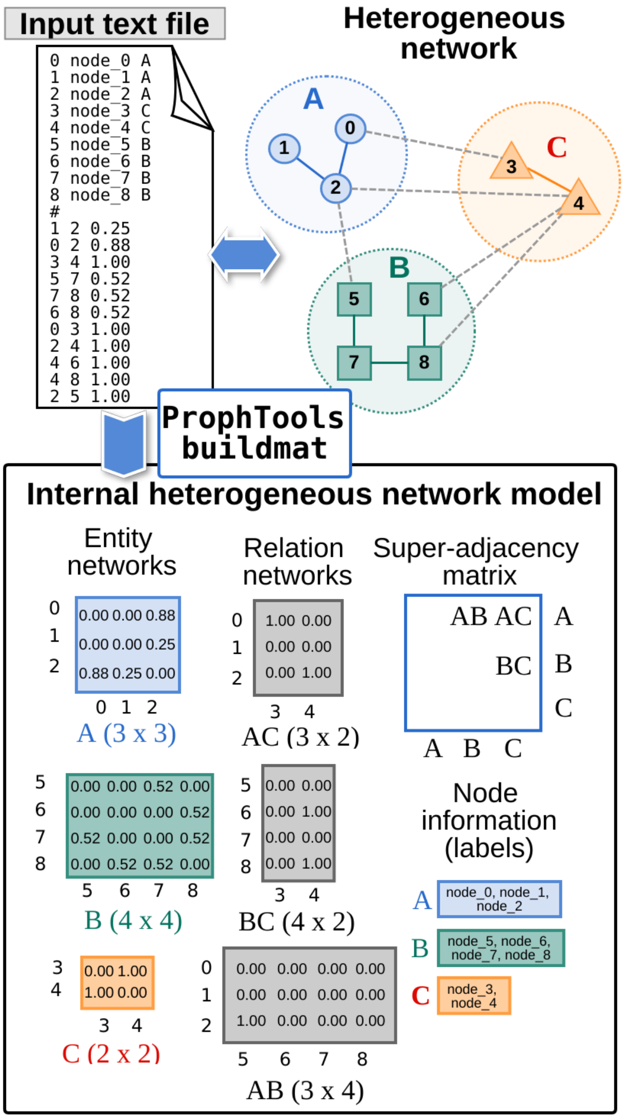

Figure 1. ProphTools heterogeneous network representation model is generated from a input text file provided by the user. This example shows a network with three types of entities: A, B, C. For each node, a numerical ID, a label and type, are specified. Edges, along with their weight, are also provided. ProphTools automatically (buildmat) converts this input to an internal representation model. For each subnetwork, an adjacency matrix is computed and normalized. Raw edge values are explicitly shown on the adjacency matrices. Additionally, connections between different entities are modelled as bipartite adjacency matrices. Finally, a super-adjacency matrix models how each relation matrix connects two entity matrices.

Due to the increased relevance that long non-coding RNAs have acquired in the scientific community in the recent years, several *in-silico* and *ad-hoc* approaches have been published to sistematically predict new relations between lncRNAs and diseases. LncRNAdisease [28] is a database including experimentally validated relations of lncRNAs and diseases and predictions based on these instances. *LRLSLDA* [28] defines a classi-

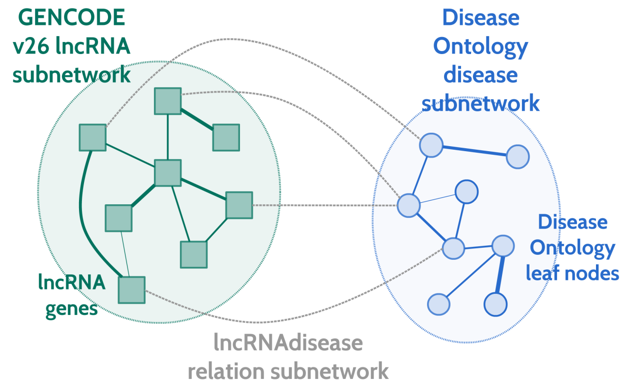

Figure 2. Heterogeneous network configuration built to perform lncRNA-disease ProphTools prioritization. LncRNA subnetwork is built from GENCODE v26 lncRNA sequences. Disease subnetwork is built from Disease Ontology leaf nodes using semantic similarity measures as in DrugNet [13]. LncRNA-disease relation subnetwork is taken from lncRNAdisease database [28]. This data configuration file is available at ProphTools website.

fication function based on the assumption that similarity between diseases can be an indicator of the similarity between lncRNAs they are associated to. Later, their authors released *IRWRLDA* [29], a network-based lncRNA-disease prioritization algorithm that uses disease semantic similarity and lncRNA expression data to relate lncRNAs, and a modification of a Random Walk with Restarts (RWR) algorithm to perform prioritization. *RWRlncD* [30] also implements RWR on a lncRNA similarity network. LncRNA similarity is also based on the disease sets each lncRNA is associated to in *lncRNAdisease* database [28]. A disadvantage of this method is that it can only perform prioritization on lncRNAs that are associated to at least one disease, which are a very small proportion (156) of the total number of lncRNAs annotated in the human genome (currently 15787 lncRNA annotations in the latest release of GENCODE). More recently, Yao *et al.* proposed *LncPriCNet* [31], a method that built a multi-level network in order to perform lncRNA-disease prioritization.

All these approaches are *ad-hoc* methods developed to solve the lncRNA-disease prioritization problem, not available as general source code. Additionally, the current lack of knowledge about lncRNAs and their relation to disease makes it probably difficult to draw conclusions about a broad set of lncRNAs, since available functional annotations are about two orders of magnitude smaller than the global amount of lncRNA candidates. Due to the interest these biological entities have drawn in the recent years, it seems very likely that this knowledge grows in the near future, and more lncRNA-disease annotations will be made available. However, users will not be able to include future knowledge in these methods, as they are not available as flexible, general-purpose tools.

Here, we apply ProphTools to lncRNA-disease prioritization, as a proof of concept. To do so, we need to model this problem to fit the proposed heterogeneous network representation. Figure 2 shows the network configuration chosen to integrate the available data on lncRNAs and diseases.

Although ProphTools has not been specifically designed to accomplish this particular problem, obtained results are consistent with the current knowledge about lncRNAs and ProphTools is proven as effective as other state-of-the art *ad-hoc* methodologies. Furthermore, the datasets built are freely available to the scientific community to ensure reproducibility and allow further research and improvements on the topic.

Data

The heterogeneous network includes two entity subnetworks: long non-coding RNAs (lncRNAs) and diseases, and a relation subnetwork lncRNA-disease connecting them (figure 2).

LncRNA network was built using GENCODE v26 [32, 33]. The 15787 lncRNA gene annotations present in GENCODE v26 were processed by generating a projection of overlapping exons for each lncRNA and building a projected transcript representative of each lncRNA. The sequence of each projected transcript was then obtained from the repeat masked version of the human genome hg38. In order to reflect the modular functionality present in lncRNAs [34], we represented each lncRNA gene as a vector of hexamers (short subsequences of 6 nucleotides length). For each lncRNA gene sequence, the appearances of each of the 4096 possible hexamers were counted. These vectors were compared to each other to build an adjacency matrix using as similarity measure the cosine similarity between the hexamer occurrence vectors. These similarities were used as edge weight in our lncRNA network. Additionally, the obtained adjacency matrix was postprocessed removing 50% of the edges, in order to remove propagation noise while keeping the whole network as a single connected element. After this process, 125 isolated nodes (lncRNA genes) were removed from the final network, which connects 15662 lncRNAs.

The disease network was obtained from the Disease Ontology, applying the same processing as described for Drugnet [13]. The resulting network includes 4517 diseases that correspond to leaf nodes in the Disease Ontology.

Finally, the lncRNA-disease network was built from lncRNAdisease database [28]. A file corresponding to 1102 experimentally-validated lncRNA-disease connections was downloaded from the lncRNAdisease website [35]. After removing duplicated connections in the lncRNA-disease file, 687 edges were obtained. Naming conventions used in this file for lncRNAs and diseases needed to be matched to GENCODE and the Disease Ontology identifiers, respectively. The ID matching process was performed using fuzzy string matching based on Levenshtein Distance [36] and results were manually revised. The lncRNA id matching process resulted in a set of 229 matches out of 377, after removing general characterizations of sets of lncRNAs (e.g. RNA polymerase III-dependent lncRNAs), and filtering for human long non-coding RNAs. The disease matching process was performed using the same fuzzy string matching library, but two correspondence files were generated: one allowing multiple matches for each lncRNAdisease identifier, and one storing only the best match. Since our disease network includes leaf nodes from the Disease Ontology, non-specific identifiers such as "cancer" or "leukemia" correspond to a set of nodes in our disease network. The multiple matches approach is thus including even more redundancy in the network. To quantify and evaluate this effect in the results, we tested with both options.

After this identifier matching process we obtained two lncRNA-disease datasets: i) a `generic` dataset, consisting of 837 relations, including multiple synonyms for generic terms such as "cancer" [see Additional File 1], and ii) a `specific` dataset, consisting of 352 relations where only the best match for the generic terms was included [see Aditional File 2]. After removing connections to isolated lncRNA nodes, the resulting datasets have 829 and 347 relations, respectively.

Results

This heterogeneous network configuration was then tested for performance using 5-fold Cross Validation. This functionality is also implemented in the ProphTools package. Since

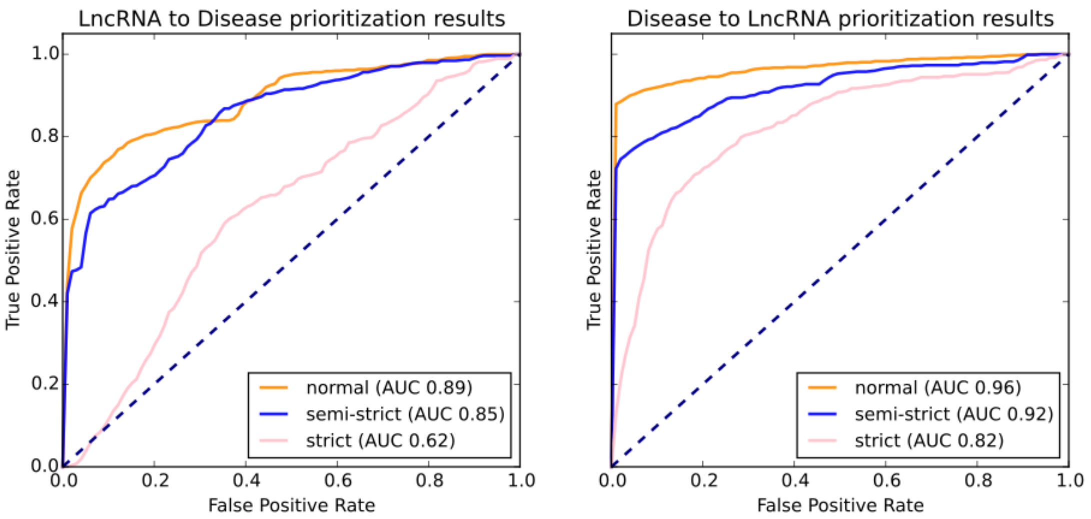

Figure 3. Results for 5-fold CV tests on the `general` dataset obtained from experimental evidence in LncRNAdisease database. On the left side, ROC curves obtained for normal, semi-strict and strict tests performed from lncRNA to disease. On the right side, ROC curves obtained for disease to lncRNA prioritization.

our disease network includes leaf nodes from the Disease Ontology, non-specific disease associations such as "cancer" or "leukemia" do not yield a single match, but a set of disease nodes. This results in a set of edges representing one correspondence in the original dataset, which could artificially improve the results in a performance test, since it would allow a certain degree of redundancy that would persist after removing the tested edge. Taking this into account, we have performed two additional versions of the CV test, namely i) a semi-strict version that removes at once all edges connecting a certain entity with the destination network in the direction of propagation, and ii) a strict version that removes all edges connecting the entities in both sides of the test edge (i.e. a semi-strict test for both directions of propagation). As figures 3 and 4 show, the AUC value is strongly affected by these two tests, specially if the direction of prioritization is lncRNA-disease (i.e. queries are lncRNAs and targets are diseases). Interestingly, if we perform a disease-lncRNA prioritization (queries are diseases and targets are lncRNAs) the semi-strict and strict CV tests have lower impact on the final results. This could be related to the Disease Ontology semantic similarity structure, that generates groups of strongly related nodes, such as families of diseases. Furthermore, the lncRNA-network is scarcely populated. The amount of edges provided by the test datasets (829 `general`, 347 `specific`) is very reduced compared to the amount of diseases and lncRNAs in the lncRNA and disease networks, and there are groups of diseases, such as cancer, that cover a high percentage of the total dataset (~ 23.66 % for the `general` dataset and ~ 18.18 for the `specific` dataset). Performing a strict test can eliminate not only the synonyms introduced in the `general` dataset, but also additional information that comes from a different source. If many of these cases occur, the resulting prioritization method tries to propagate from one network to another through very few connecting interactions, resulting in poor correlation scores. We believe this effect would be alleviated by a more populated lncRNA-disease network. However, it is interesting to note that although the strict test performs poorly for lncRNA-disease prioritization, the semi-strict test results are not affected by the removal of synonyms.

Normal, semi-strict and strict results are reported in table 1. Normal tests show a 0.963 ± 0.008 AUC value for lncRNA-disease prioritization and a 0.888 ± 0.015 AUC for disease-lncRNA prioritization for the `general` dataset (see table 1), and a 0.850 ± 0.030 AUC value for lncRNA-disease prioritization and a 0.886 ± 0.012 AUC for disease-lncRNA prioritization for the `specific` dataset (see table 2). These results show that predictions made by ProphTools with the proposed heterogeneous network configuration are consistent with current knowledge about lncRNAs and diseases and therefore likely to provide new predictions of interest. These AUC values are competitive with state-of-the art *ad-hoc* approaches, such as *IRWRLDA* [29] (0.7242 and 0.7872 AUC values), *LRLSLDA* [37] (0.7760 AUC value), and *RWRlncD* [30] (0.822 AUC value), and the recent *LncPriCNet* [31] (0.93 AUC value). Furthermore, single prioritization queries on the `specific` dataset ran on average between 8.14 (± 0.04) seconds for lncRNA-disease prioritization and 11.86 (± 0.52) seconds for disease-lncRNA on our server[1]. The same test ran on average between 11.65 (± 1.29) seconds for lncRNA-disease prioritization and 14.61 (± 5.16) seconds for disease-lncRNA on a laptop[2].

Conclusions

ProphTools is an open-source, flexible, modular and easy-to-use general implementation of an heterogeneous propagation algorithm that has been proven useful for relevant applications such as gene-disease prioritization and drug repositioning. The abstraction data layer we provide allows users to run ProphTools in any dataset of interest. As a proof of these features, a case study on lncRNA-disease prioritization has been described. Results are competitive with state-of-the art approaches in the field. In order to ensure the reproducibility of the results and allow further improvements in lncRNA-disease prioritization, the datasets built to apply ProphTools have also been made available.

ProphTools source code is available both as a GitHub repository and as a standalone python package that can be easily installed via pip [19] and also run as a Docker container [20]. Additionally, ProphTools is not only open-source but also very modular in design, allowing advanced users to extend its functionality. We are already working on further features (such as additional propagation algorithms) to incorporate to the ProphTools framework in future versions. Although preprocessing

1 Intel(R) Xeon(R) CPU E5-2680 v3 @ 2.50GHz (x48), 256GiB RAM
2 Dell XPS 13, Intel(R) Core(TM) i7-5500U CPU @ 2.40GHz, 8GiB RAM

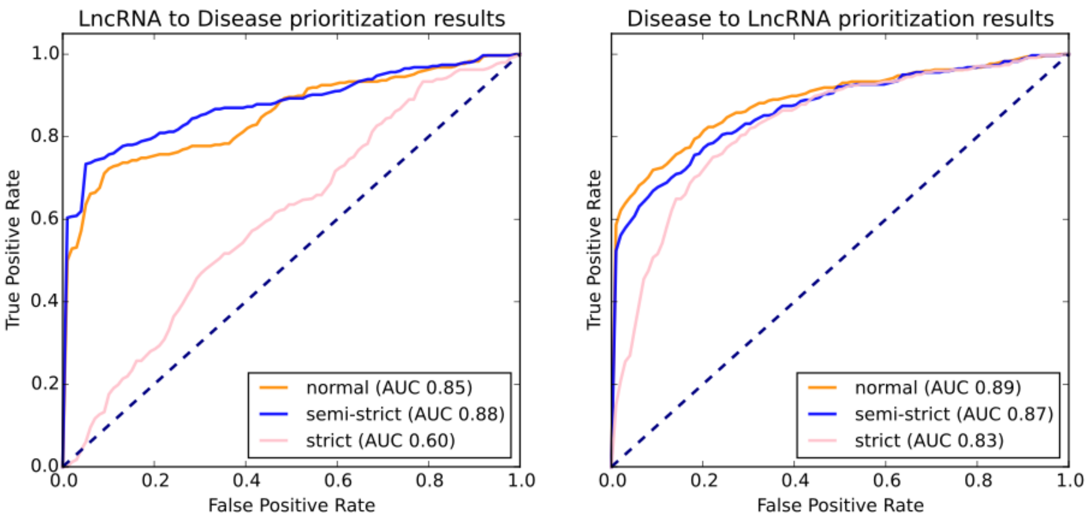

Figure 4. Results for 5-fold CV tests on the `specific` dataset obtained from experimental evidence in LncRNAdisease database. On the left side, ROC curves obtained for the normal, semi-strict and strict test performed from lncRNA to disease. On the right side, ROC curves obtained for disease to lncRNA prioritization.

Table 1. ProphTools performance results on the specific and general lncRNA-disease datasets for three different 5-fold CV modes. Normal CV removes only one edge per test. Semi-strict test mode removes all edges including origin nodes in the test set towards propagation direction, and strict mode removes all edges involving the two nodes connected by each test edge in the test set. Propagation direction shows whether lncRNAs or diseases are are being ranked. Mean AUC column shows the average AUC obtained at the 5-fold CV test for each category. Mean Rank shows the average ranking obtained for each test case, and Mean Rank% shows the mean rank as percentage.

Dataset	CV test mode	Propagation direction	Mean AUC		Mean Rank		Mean Rank %	
General	normal	lncRNA	0.963	± 0.008	522.16	± 120.21	3.33	± 0.77
		disease	0.888	± 0.015	503.67	± 69.57	11.15	± 1.54
	semi-strict	lncRNA	0.917	± 0.008	1240.95	± 130.76	7.92	± 0.83
		disease	0.854	± 0.018	655.17	± 78.45	14.50	± 1.74
	strict	lncRNA	0.823	± 0.016	2767.30	± 241.71	17.67	± 0.01
		disease	0.618	± 0.096	1725.18	± 435.05	38.19	± 9.63
Specific	normal	lncRNA	0.886	± 0.012	1751.24	± 194.68	11.18	± 1.24
		disease	0.850	± 0.030	670.48	± 135.53	14.84	± 3.00
	semi-strict	lncRNA	0.866	± 0.026	2059.37	± 403.71	13.15	± 2.58
		disease	0.877	± 0.020	548.05	± 91.76	12.13	± 2.03
	strict	lncRNA	0.828	± 0.034	2690.47	± 476.54	17.18	± 3.04
		disease	0.602	± 0.058	1794.22	± 261.58	39.72	± 5.79

work is required in order to build a network model, we believe ProphTools flexible representation of heterogeneous networks and its support for different input file formats reduces the amount of work required to perform analyses in an *ad-hoc* manner.

We expect that the availability of our prioritization method as an open-source, customizable tool can be of use for a wide range of biological applications.

Availability of supporting source code and requirements

- Project name: ProphTools.
- Project home page: https://github.com/cnluzon/prophtools, https://hub.docker.com/r/cnluzon/prophtools/
- Operating systems: Linux, platform independent if using the Docker version.
- Programming language: Python 2.7.
- Other requirements: Non-linux systems need to run the Docker version. Native linux systems require following python libraries (installed automatically when installing via pip): numpy (>=1.11.2), scipy (>=0.18.1), matplotlib (>=1.4.3), scikit-learn (>=0.18), networkx (>=2.0).
- SciCrunch RRID (Research Resource Identification Initiative ID): SCR_015813.
- License: GNU GPLv3.0.

Availability of supporting data and materials

ProphTools source code available at GitHub [19] and as a Docker container at Docker hub [20]. Heterogeneous network configurations for lncRNA-disease prioritization are available for download at ProphTools website [15]. Drug-gene-disease prioritization data is also available at our server [14]. An archival copy of the code and other supporting data are available via the Gigascience repository, GigaDB [38].

Declarations

List of abbreviations

- lncRNA. Long non-coding RNA.
- RWR. Random Walk with Restarts.
- CV. Cross-Validation.

Ethics approval and consent to participate

Not applicable.

Consent for publication

Not applicable

Competing interests

The authors declare that they have no competing interests.

Funding

This work was supported by Dirección General de Investigación Científica y Técnica [TIN2013-41990-R and DPI2017-84439-R]; European Regional Development Fund; and Spanish Ministry of Education, Culture and Sports [C. Navarro's FPU grant].

Authors' contributions

CN developed the software, both the python repository and the Dockerized version, performed data analysis and wrote the paper. VM developed the methodology and participated in the core software functionality. CC supervised the development of the software and data analysis, reviewed and edited the paper. AB conceptualized the research idea, supervised the quality of the process and also reviewed and edited the draft.

Acknowledgements

Not applicable.

References

1. Hu JX, Thomas CE, Brunak S. Network biology concepts in complex disease comorbidities. Nature Reviews Genetics 2016;.
2. Chasman D, Siahpirani AF, Roy S. Network-based approaches for analysis of complex biological systems. Current opinion in biotechnology 2016;39:157–166.
3. Hu Y, Zhang Y, Ren J, Wang Y, Wang Z, Zhang J. Statistical approaches for the construction and interpretation of human protein-protein interaction network. BioMed research international 2016;2016.
4. Lotfi Shahreza M, Ghadiri N, Mousavi SR, Varshosaz J, Green JR. A review of network-based approaches to drug repositioning. Briefings in Bioinformatics 2017;p. bbx017.
5. Layeghifard M, Hwang DM, Guttman DS. Disentangling interactions in the microbiome: a network perspective. Trends in microbiology 2017;25(3):217–228.
6. Valentini G, Armano G, Frasca M, Lin J, Mesiti M, Re M. RANKS: a flexible tool for node label ranking and classification in biological networks. Bioinformatics 2016;p. btw235.
7. Franceschini A, Lin J, von Mering C, Jensen LJ. SVD-phy: improved prediction of protein functional associations through singular value decomposition of phylogenetic profiles. Bioinformatics 2015;p. btv696.
8. Blatti C, Sinha S. Characterizing gene sets using discriminative random walks with restart on heterogeneous biological networks. Bioinformatics 2016;p. btw151.
9. Pathan M, Keerthikumar S, Ang CS, Gangoda L, Quek CY, Williamson NA, et al. FunRich: An open access standalone functional enrichment and interaction network analysis tool. Proteomics 2015;15(15):2597–2601.
10. Moreau Y, Tranchevent LC. Computational tools for prioritizing candidate genes: boosting disease gene discovery. Nature Reviews Genetics 2012;13(8):523–536.
11. Isik Z, Baldow C, Cannistraci CV, Schroeder M. Drug target prioritization by perturbed gene expression and network information. Scientific reports 2015;5.
12. Martínez V, Cano C, Blanco A. ProphNet: A generic prioritization method through propagation of information. BMC bioinformatics 2014;15(1):1.
13. Martínez V, Navarro C, Cano C, Fajardo W, Blanco A. DrugNet: Network-based drug–disease prioritization by integrating heterogeneous data. Artificial intelligence in medicine 2015;63(1):41–49.
14. Navarro C, Martínez V, Cano C, Blanco A, Drug-gene-disease prioritization prioritization heterogeneous network configuration supplementary material.;. http://genome.ugr.es:9000/download/data/drugnet_np.zip. Accessed September 18, 2017.
15. Navarro C, Martínez V, Cano C, Blanco A, LncRNA-disease prioritization heterogeneous network configuration supplementary material: specific and general datasets.;. http://genome.ugr.es:9000/download/data/prophtools_gn.zip; http://genome.ugr.es:9000/download/data/prophtools_sp.zip. Accessed September 18, 2017.
16. Hwang T, Zhang W, Xie M, Liu J, Kuang R. Inferring disease and gene set associations with rank coherence in networks. Bioinformatics 2011;27(19):2692–2699.
17. Pedregosa F, Varoquaux G, Gramfort A, Michel V, Thirion B, Grisel O, et al. Scikit-learn: Machine learning in Python. Journal of Machine Learning Research 2011;12(Oct):2825–2830.
18. Travis CI Continuous Integration platform;. https://travis-ci.org/. Accessed September 18, 2017.
19. Navarro C, Martínez V, Cano C, Blanco A, ProphTools: General Prioritization Tools for Heterogeneous Biological Networks.; 2017. GitHub repository. http://www.github.com/cnluzon/prophtools. Accessed September 18, 2017.
20. Navarro C, Martínez V, Cano C, Blanco A, ProphTools: General Prioritization Tools for Heterogeneous Biological Networks.;. Docker Container. https://hub.docker.com/r/cnluzon/prophtools/. Accessed September 18, 2017.
21. GEXF file format specification;. https://gephi.org/gexf/format/.
22. Bastian M, Heymann S, Jacomy M, et al. Gephi: an open source software for exploring and manipulating networks. Icwsm 2009;8:361–362.
23. Hangauer MJ, Vaughn IW, McManus MT. Pervasive transcription of the human genome produces thousands of previously unidentified long intergenic noncoding RNAs. PLoS Genet 2013;9(6):e1003569.
24. Gibb EA, Brown CJ, Lam WL. The functional role of long non-coding RNA in human carcinomas. Molecular cancer 2011;10(1):1.
25. Iyer MK, Niknafs YS, Malik R, Singhal U, Sahu A, Hosono Y, et al. The landscape of long noncoding RNAs in the human transcriptome. Nature genetics 2015;47(3):199–208.
26. Ponting CP, Oliver PL, Reik W. Evolution and functions of long noncoding RNAs. Cell 2009;136(4):629–641.
27. Wapinski O, Chang HY. Long noncoding RNAs and human disease. Trends in cell biology 2011;21(6):354–361.
28. Chen G, Wang Z, Wang D, Qiu C, Liu M, Chen X, et al. LncRNADisease: a database for long-non-coding RNA-associated diseases. Nucleic acids research 2013;41(D1):D983–D986.
29. Chen X, You ZH, Yan GY, Gong DW. IRWRLDA: improved random walk with restart for lncRNA-disease association prediction. Oncotarget 2016;7(36):57919.
30. Sun J, Shi H, Wang Z, Zhang C, Liu L, Wang L, et al. Inferring novel lncRNA-disease associations based on a random walk model of a lncRNA functional similarity network. Molecular BioSystems 2014;10(8):2074–2081.

31. Yao Q, Wu L, Li J, guang Yang L, Sun Y, Li Z, et al. Global Prioritizing Disease Candidate lncRNAs via a Multi-level Composite Network. Scientific Reports 2017;7.
32. Derrien T, Johnson R, Bussotti G, Tanzer A, Djebali S, Tilgner H, et al. The GENCODE v7 catalog of human long noncoding RNAs: analysis of their gene structure, evolution, and expression. Genome research 2012;22(9):1775–1789.
33. GENCODE Project Homepage;. http://gencodegenes.org. Accessed September 18, 2017.
34. Mercer TR, Mattick JS. Structure and function of long noncoding RNAs in epigenetic regulation. Nature structural & molecular biology 2013;20(3):300–307.
35. Chen G, Wang Z, Wang D, Qiu C, Liu M, Chen X, et al., LncRNADisease: a database for long-non-coding RNA-associated diseases (website).;. http://www.cuilab.cn/lncrnadisease. Accessed September 18, 2017.
36. Fuzzy Wuzzy fuzzy string matching python library;. https://github.com/seatgeek/fuzzywuzzy. Accessed September 18, 2017.
37. Chen X, Yan GY. Novel human lncRNA–disease association inference based on lncRNA expression profiles. Bioinformatics 2013;p. btt426.
38. Navarro C, Martínez V, Blanco A, Cano C, Supporting data for "ProphTools: General Prioritization Tools for Heterogeneous Biological Networks". GigaScience Database; 2017. http://dx.doi.org/10.5524/100369.

Figure 1

Click here to download Figure toy_example_fig_vertical.png

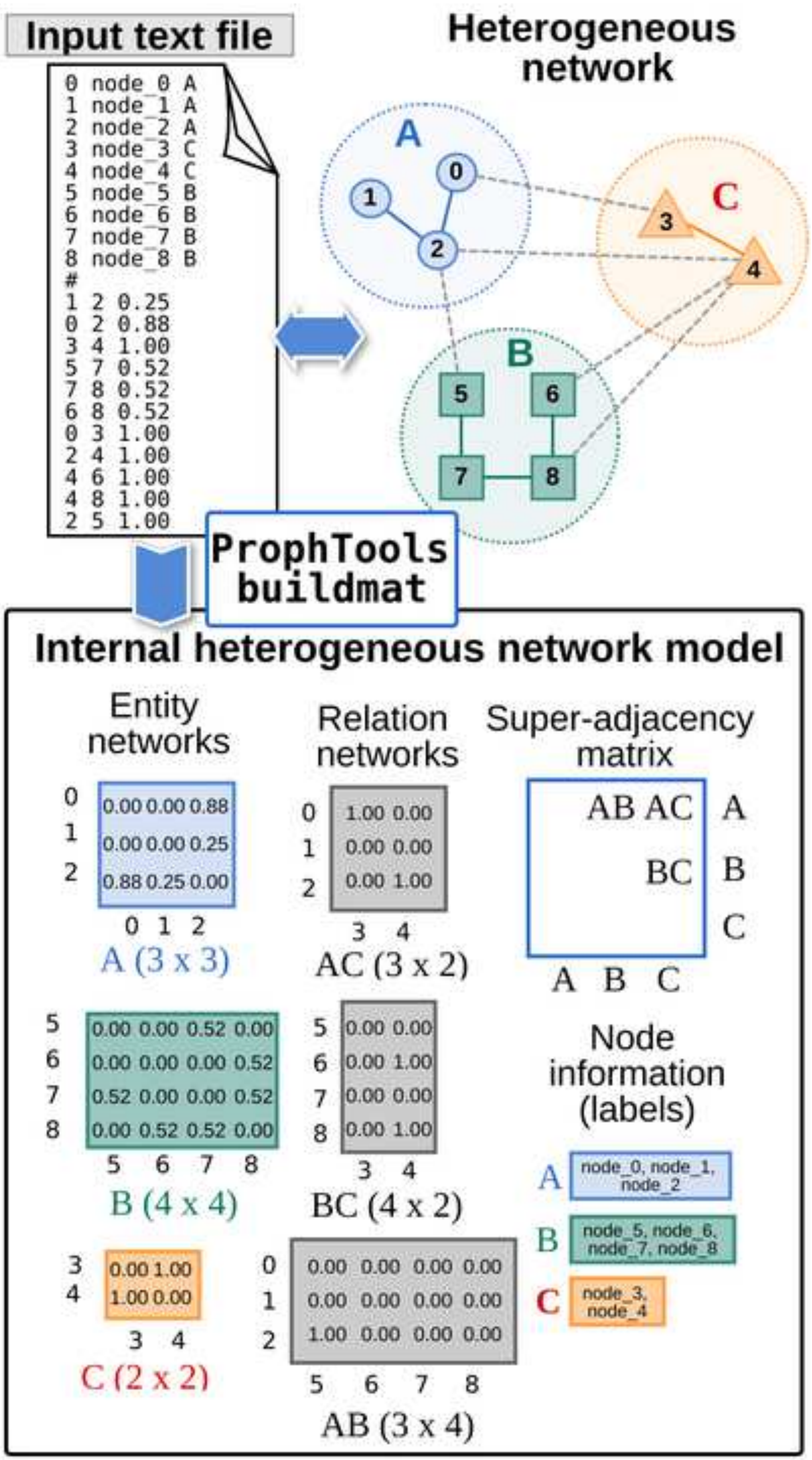

Figure 2

Click here to download Figure fig02_lncrnadisease.png

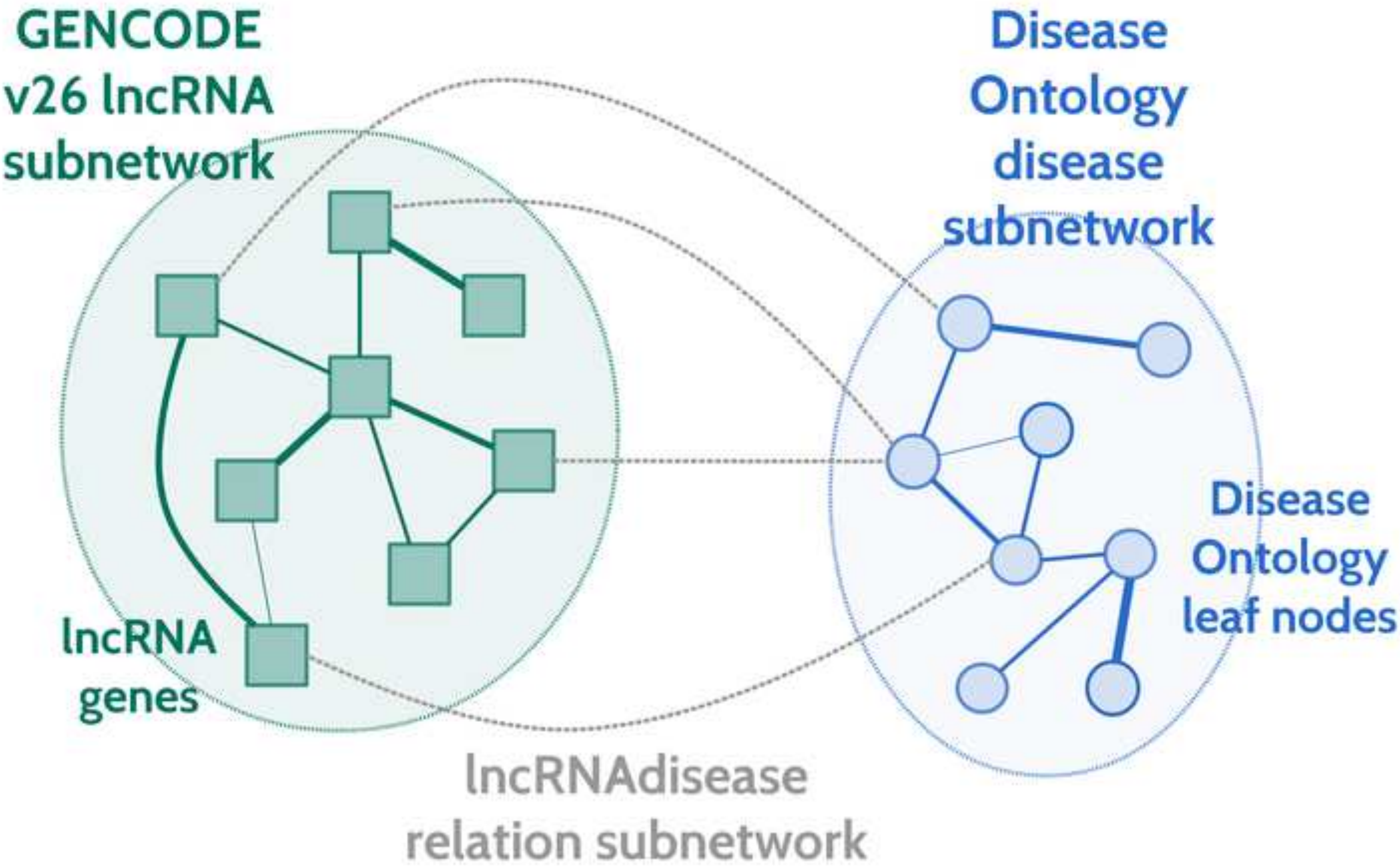

Figure 3

Click here to download Figure fig03_lootests.png

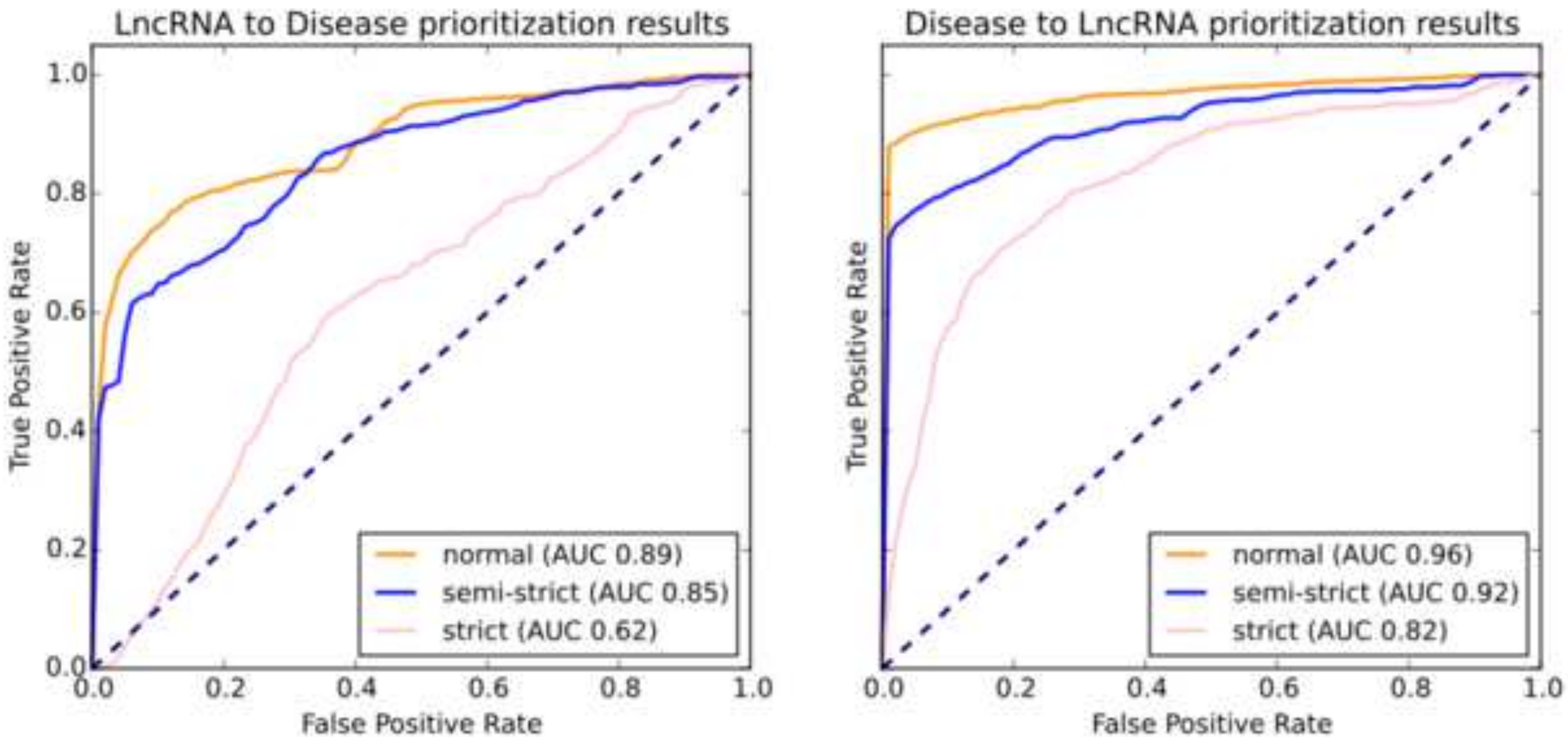

Figure 4

Click here to download Figure fig04_lootestsspecific.png

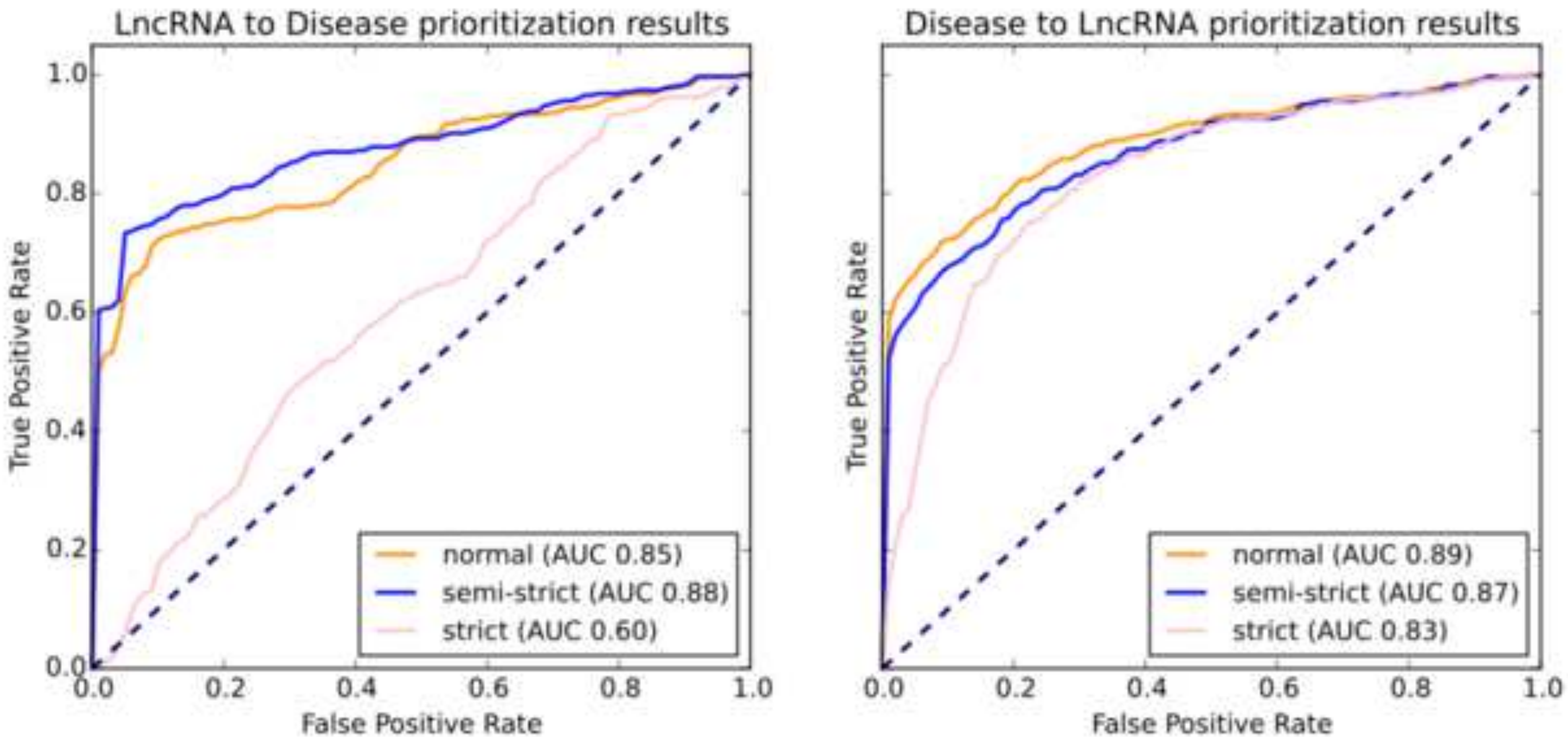

Additional File 1. General dataset.

Click here to access/download

Supplementary Material

SupFile01_lncrna_disease_prophtools_general.csv

Additional File 1 description file.

Click here to access/download

Supplementary Material

SupFile01_lncrna_disease_prophtools_general_info.txt

Additional File 2. Specific dataset.

Click here to access/download
Supplementary Material
SupFile02_lncrna_disease_prophtools_specific.csv

Additional File 2 description file.

Click here to access/download
Supplementary Material
SupFile02_lncrna_disease_prophtools_specific_info.txt

Main LaTeX manuscript source

Click here to access/download
Supplementary Material
main.tex

References file for manuscript source

Click here to access/download
Supplementary Material
paper-refs.bib

LaTex style files

Click here to access/download
Supplementary Material
oup-contemporary.cls

Click here to access/download
Supplementary Material
vancouver-authoryear.bst

Logo required in the main.tex

Click here to access/download
Supplementary Material
gigascience-logo.pdf

Logo required in the main.tex

Click here to access/download
Supplementary Material
oup.pdf
