# Supplementary material for: ProphTools: general prioritization tools for heterogeneous biological networks
Source: Gigascience. 2017 Nov 24;6(12):1–8. doi: 10.1093/gigascience/gix111 (PMC5751048; doi:10.1093/gigascience/gix111)
Supplement: Response-to-Reviewer-Comments_Original-Submission.pdf [file gix111_response-to-reviewer-comments_original-submission.pdf]

Author's Response To Reviewer Comments

Dear Editor,

please find our revised manuscript, now adapted to the journal style, and our response to reviews in this new submission. We are very thankful for the insights provided and the detailed feedback. All the comments have been thoroughly considered and several changes have been made in order to improve the manuscript according to these suggestions. Furthermore, we have extended the functionality of the proposed tool in order to address the usability concerns of reviewer #2. We believe that ProphTools has greatly benefitted from these reviews.

As requested, each comment from the reviewers is addressed below in a point-by-point manner. We hope these improved versions of manuscript and method are suitable for their publication in GigaScience.

Sincerely,

- Authors

GIGA-D-17-00123
ProphTools: General Prioritization Tools for Heterogeneous Biological Networks
Carmen Navarro Luzón; Víctor Martínez Gómez; Armando Blanco Morón; Carlos Cano Gutiérrez
GigaScience

Dear Ms. Navarro Luzón,

Your manuscript "ProphTools: General Prioritization Tools for Heterogeneous Biological Networks" (GIGA-D-17-00123) has been assessed by our reviewers. Although it is of interest, we are unable to consider it for publication in its current form. The reviewers have raised a number of points which we believe would improve the manuscript and may allow a revised version to be published in GigaScience.

Their reports, together with any other comments, are below.

I'm happy to say that reviewer 1 is quite positive regarding your paper and requires only a few rather minor revisions.

Reviewer 2 is also positive overall, however, the reviewer is not quite convinced that it is really that easy to run the tool "out of the box", as claimed in the manuscript. In light of the reviewer's comments, you may decide to add some minor improvements to the tool and/or describe preprocessing steps etc. in more detail, to appeal to a wider user base.

If you are able to fully address both reviewers' points, we would encourage you to submit a

revised manuscript to GigaScience. Once you have made the necessary corrections, please submit online at:

http://giga.edmgr.com/

If you have forgotten your username or password please use the "Send Login Details" link to get your login information. For security reasons, your password will be reset.

Please include a point-by-point within the 'Response to Reviewers' box in the submission system. Please ensure you describe additional experiments that were carried out and include a detailed rebuttal of any criticisms or requested revisions that you disagreed with. Please also ensure that your revised manuscript conforms to the journal style, which can be found in the Instructions for Authors on the journal homepage.

The due date for submitting the revised version of your article is 02 Oct 2017.

I look forward to receiving your revised manuscript soon.

Best wishes,

Hans Zauner
GigaScience
www.gigasciencejournal.com

Reviewer reports:

Reviewer #1: In this paper the authors propose a very general prioritization tool for the analysis of heterogeneous biological networks, by which it is possible to prioritize genes or any other biomolecular or biological entity with respect to given concepts (classes).

Differently from tools available in literature for prioritization tasks with biological networks, the proposed approach is able to integrate and exploit the "relational knowledge" embedded in heterogeneous networks (i.e. networks whose nodes represents different biological entities) to discover new associations between biological entities lying in different networks.
This is accomplished through a propagation of the information not only within each component homogeneous networks of the overall heterogeneous network system, but also across different networks, by adopting a transductive semi-supervised learning approach.
This is a very new algorithmic approach that can be applied to a wide range of network-based problems in computational biology, ranging from protein function prediciton, to disease-gene prioritization and drug repositioning.
Due to the flexibility of this tool, ProphTools can be applied also to novel prioritization problems, as clearly and conivincingly shown by the proof-of-concept application of ProphTools to long non coding RNA (lncRNA) - disease prioritization. Moreover this approach can also easily integrate different types of biomolecular networks and/or biological concepts to easily exploit the knowledge available about a specific problem.
The authors provide a easy-to-use open source Python implementation of their method that could

be a valuable resource for researcher working in different areas of molecular biology and medicine.
Summarizing ProphTools provides new features not available in state-of-the-art methods: its novel methodological approach and the modularity and flexibility of its implementation makes this tool an excellent candidate for a large range of analyses in complex heterogeneous biological networks.

This is a quite clear and well-written paper, and there are only some minor issues that should be addressed by the authors:

1. The authors should better explain what are the characteristics of the super-adjacency matrix introduced in Section 2.1. For instance, they could provide a simple "toy" example or a more formal representation of it.

We included specific information on the contents of the adjacency matrices and the super-adjacency matrix in a new version of Figure 1, depicting a toy example.
In addition, ProphTools now also supports two new formats for the input heterogeneous network provided by the user: a plain text format and Graph Exchange XML File (GEXF) format. These two formats are more intuitive than the native internal representation model, making ProphTools easier to apply to new domains. All these formats are thoroughly described in the documentation. An example using the plain text format has also been included in Figure 1. The description of ProphTools internal heterogeneous network representation model has also been kept in the text for clarity.

2. The authors should provide more details about the ProphTools prioritization methodology. For instance, even if the weighted across-network propagation algorithm has been explained in detail in their previous cited work [8], it would be helpful to introduce some more details about this algorithm (e.g. in the Section 2.1) to make the paper more self-contained and easier to be read.

We have included the pseudocode for the algorithm and some further details on the “Approach” section. Details about the representation model or how to run the tool have been moved to a “How to use” section to improve the readability of the manuscript.

3. The results about the application of lncRNA-disease prioritization are clear and convincing, but the authors should better explain what they mean for Leave-One-Out with 5-fold cross-validation. Even if I can guess what they could mean with this expression, this is not standard terminology, since strictly speaking leave-one-out is a particular case of cross-validation in which each single example is left out for testing.

We agree this terminology might lead to confusion. We have replaced “Leave One Out with Cross Validation” with “Cross Validation” only, since LOO is a particular case of Cross Validation and we are testing for absence of one fifth of the edges in each test set (for a five-fold cross validation), to better test the predictive ability of our network configuration.

4. It would be nice also if the authors could provide some information about the empirical time

complexity of their approach (e.g. with respect to the considered proof-of-concept application to lncRNA-disease prioritization).

Thanks for pointing out this issue. As suggested, we have included a comment in the new version of the manuscript on average runtimes for the proposed case study on lncRNA-disease prioritization.

5. I suggest to merge Table 1 and Table 2, since they have exactly the same caption (apart from the title in bold)

Both tables have now been merged in one larger table with a single caption.

Reviewer #2: The authors have packaged their algorithm and previous work into a tool for usage by broader research community. Hence, while the algorithm and its efficacy is established, the usage of this tool for a generic applicability and its usability/user-friendly aspect can be improved.

All the line numbers mentioned below refer to the original manuscript's line numbers

Technical:
1) Only because the authors have positioned ProphTools as a "general, ready to use", "can be used out-of-the-box" tool, it gives an impression that the tool is ready to be downloaded, plugged-and-played. However, in my estimation, this is not the case. For e.g.,
a) Generally such tools would allow for the input to be a .txt or .csv file with minimal restrictions on the input type/nature and minimal preprocessing of the input. For e.g. Girvan-Newman's community detection command line tool, or GP-Dream network inference tools, etc. ProphTools "requires" the input to be in a .mat file which requires additional familiarity with either Matlab or Python and a significant amount of input-preprocessing (e.g. adjacency matrixes, precomputed matrices, required meta-data). What's a precomputed matrix? What should it contain?

We strongly agree. It is true that generating a .mat file requires familiarity with specific programming languages and a significant amount of work for the user. This may have passed unnoticed to us due to our familiarity with these languages/methodology.
In order to address this concern, we have modified ProphTools to accept two additional file formats to represent the input networks: a plain text representation, containing only a list of nodes and edges, and GEXF (Graph exchange XML files). These formats are thoroughly described in the ProphTools online documentation, together with some examples. A toy example using the plain text format is also depicted in Figure 1. These formats only require the user to assign each node a numeric code, a label and a type (the subnetwork it belongs to). The rest of the model (native .mat files, including precomputed matrices) is automatically generated by ProphTools (new functionality called buildmat). Therefore, using this new version of ProphTools, users are no longer required to manually build these mat files nor to know specific details of the implementation (e.g. how to build precomputed matrices).

b) The "How to use" instructions can be described better, step-by-step from creating a .mat file and its necessary components and formats.

We have moved the instructions about ProphTools usage to a new subsection titled "How to use". This section has been expanded with a description of the new functionality and a very simple use example: creating a mat file from a text file and performing a prioritization query using the provided network representation.

c) "The generality of ProphTools prioritization requires you to provide some meta-data along with the adjacency matrices for the entities and the relations involved in your network configuration." - Where/when should the user provide this meta-data, because in the instructions on usage, the command is "prophtools prioritize --matfile network.mat --src 0 --dst 2 --query 1,2" and the input network.mat file does not have any meta-data as described in the "README.rst".

We apologise for the lack of precision with the term "meta-data". With "meta-data" we referred here to the data in ProphTools' heterogeneous network representation model that did not relate to the edge values or nodes, but information about the subnetworks or their relationships (i.e. the super-adjacency matrix). This has been rephrased in the manuscript to avoid any confusion, as the new input data types simplify this process.

2) In the motivation, the authors have emphasized in their writing, figures and example code about the application of ProphTools to heterogenous networks of at least 3 sub-networks. However, in the case study, they have implemented ProphTools only 2 sub-networks (lncRNAs and diseases). It would certainly be more convincing with an application of at least 3 subnetworks, especially since its written on line 105 "For instance, if a drug-target-disease network configuration is provided, ProphTools' ability to predict drug-disease relations can be estimated by performing a 5-fold LOO-CV test on such relation."

Adding a new type of entity (subnetwork) to an existing network representation requires the availability of data sources to connect entities of this type and to other data types (subnetworks). Unfortunately, the available sources of data on lncRNAs are not numerous nor extensive yet, as these entities are still poorly known. For this reason, it was difficult to build a meaningful three-subnetwork model for this problem in particular. In the manuscript, we wanted not only to point out the ability of ProphTools to integrate multiple subnetworks, which has already been proven in previous applications, such as gene-domain-disease prioritization (Ref. [12] in the paper), but also its generality and adaptability to a new problem. We also believed that the lncRNA-disease prioritization problem was of particular impact now, as lncRNAs have been attracting increasing attention in the scientific community.

3)The amount of preprocessing required (in addition to familiarity with Matlab/Python) is quite substantial and hence, if these tasks/datasets can be automated then it makes ProphTools more user-friendly and ready to use as an out-of-box utility.

We have expanded ProphTools functionality to support more user-friendly file formats which require less preprocessing and no familiarity with Matlab/Python. Furthermore, we have

modified the manuscript to clarify the scope of ProphTools.

3) In general, this work lacks substantial motivation - on line 67, it says "ProphTools is based on an approach that has been proven useful in several prioritization applications, such as gene-disease prioritization[8] and drug repositioning[9]". The cited references are the authors' previous works. Hence, a broader literature review describing the diverse applicability and potency of the prioritization method is needed.

The motivation of the proposed work was to provide an open source methodology that could be easily used by a broad set of users for their own problem domains. In this sense, ProphTools' core prioritization method had already been described and applied successfully in previous works by the authors to other biological fields of interest.
However, we understand that we might broaden the literature review and point out more application domains and prioritization methods. We have modified the Background section to include more fields of application and citations of recent works on such fields. Further, the introduction to the case study on lncRNA-disease prioritization also reviews the latest prioritization methods applied to this area, and the Results section includes their average accuracy in comparison to that obtained by ProphTools.

4) The case study is good and certainly highlights the usability of the tool; however, the significant preprocessing performed here underscores my aforementioned concern of "ready-to-use and out-of-the-box" ease of usage in #1.

Again, we agree with the reviewer in his concern about the amount of work required to build the lncRNA-disease network to run ProphTools. Although our goal is to reduce the complexity of network inference by providing an easy to use, general-purpose tool, users still need to preprocess different data sources in order to build their networks. In this sense, there is part of this preprocessing work which is out of the scope of the proposed method.
We have tried to clarify this point in the new version of the manuscript, in order to provide a more precise idea about the scope of ProphTools. Among other modifications, we have for example removed the references to the tool being "out-of-the-box" or "ready-to-use" (easy-to-use instead) in order not to provide the reader with misleading impressions on the scope or potential of ProphTools.

Non-technical:
1) Typos can be corrected.
- Line 30, "...as nodes in a graph, *whereas* weighted edges correspond to interactions or any type of relationship...". Should be "where" and not "whereas".
- Line 43, "RANKS[2] performs node prioritization on some label *o* property by using kernelized score functions". "of" ?
- Line 117, "systematically" has been misspelled as "sistematically".
- Case study and not "Case of study".
- Lines 257-261 repeat the information earlier stated and are not necessary.

These errors have been corrected in the new version of the manuscript.

The authors have done a commendable job in making their code, data easily available, reproducible and abided by good programming standards of software engineering and code distribution.

We are glad the reviewer found our coding and data availability efforts commendable. All the comments are appreciated.
