# Supplementary material for: ProphTools: general prioritization tools for heterogeneous biological networks
Source: Gigascience. 2017 Nov 24;6(12):1–8. doi: 10.1093/gigascience/gix111 (PMC5751048; doi:10.1093/gigascience/gix111)
Supplement: Response-to-Reviewer-Comments_Revision-1.pdf [file gix111_response-to-reviewer-comments_revision-1.pdf]

## Author's Response To Reviewer Comments

Dear editor,

please find the revised version of the manuscript in this submission. The supporting GigaDB dataset has been added to the references and mentioned in the "Availability of supporting data" section.

Source latex files are now attached and have been revised for possible typos.

The proposed tool has also been registerd at SciCrunch and the received ID displayed at "Availability of supporting source code and requirements" section.

Thank you very much for your comments and suggestions.
