# Supplementary material for: ProphTools: general prioritization tools for heterogeneous biological networks
Source: Gigascience. 2017 Nov 24;6(12):1–8. doi: 10.1093/gigascience/gix111 (PMC5751048; doi:10.1093/gigascience/gix111)
Supplement: Reviewer-1-Report-Original-Submission).pdf [file gix111_reviewer-1-report-original-submission).pdf]

Reviewer Report

Title: ProphTools: General Prioritization Tools for Heterogeneous Biological Networks

Version: Original Submission **Date:** 6/2/2017

Reviewer name: Giorgio Valentini

Reviewer Comments to Author:

In this paper the authors propose a very general prioritization tool for the analysis of heterogeneous biological networks, by which it is possible to prioritize genes or any other biomolecular or biological entity with respect to given concepts (classes).Differently from tools available in literature for prioritization tasks with biological networks, the proposed approach is able to integrate and exploit the "relational knowledge" embedded in heterogeneous networks (i.e. networks whose nodes represents different biological entities) to discover new associations between biological entities lying in different networks.This is accomplished through a propagation of the information not only within each component homogeneous networks of the overall heterogeneous network system, but also across different networks, by adopting a transductive semi-supervised learning approach.This is a very new algorithmic approach that can be applied to a wide range of network-based problems in computational biology, ranging from protein function prediciton, to disease-gene prioritization and drug repositioning.Due to the flexibility of this tool, ProphTools can be applied also to novel prioritization problems, as clearly and conivincingly shown by the proof-of-concept application of ProphTools to long non coding RNA (lncRNA) - disease prioritization. Moreover this approach can also easily integrate different types of biomolecular networks and/or biological concepts to easily exploit the knowledge available about a specific problem.The authors provide a easy-to-use open source Python implementation of their method that could be a valuable resource for researcher working in different areas of molecular biology and medicine.Summarizing ProphTools provides new features not available in state-of-the-art methods: its novel methodological approach and the modularity and flexibility of its implementation makes this tool an excellent candidate for a large range of analyses in complex heterogeneous biological networks.This is a quite clear and well-written paper, and there are only some minor issues that should be addressed by the authors:1. The authors should better explain what are the characteristics of the super-adjacency matrix introduced in Section 2.1. For instance, they could provide a simple "toy" example or a more formal representation of it.2. The authors should provide more details about the ProphTools prioritization methodology. For instance, even if the weighted across-network propagation algorithm has been explained in detail in their previous cited work [8], it would be helpful to introduce some more details about this algorithm (e.g. in the Section 2.1) to make the paper more self-contained and easier to be read.3. The results about the application of lncRNA-disease prioritization are clear and convincing, but the authors should better explain what they mean for Leave-One-Out with 5-fold cross-validation. Even if I can guess what they could mean with this expression, this is not standard terminology, since strictly speaking leave-one-out is a particular case of cross-validation in which each single example is left out for testing.4. It would be nice also if the authors could provide some information about the empirical time complexity of their approach (e.g. with respect to the

considered proof-of-concept application to lncRNA-disease prioritization).5. I suggest to merge Table 1 and Table 2, since they have exactly the same caption (apart from the title in bold)

Level of Interest

Please indicate how interesting you found the manuscript: An article of importance in its field

Quality of Written English

Please indicate the quality of language in the manuscript: Acceptable

Declaration of Competing Interests

Please complete a declaration of competing interests, considering the following questions:

- Have you in the past five years received reimbursements, fees, funding, or salary from an organisation that may in any way gain or lose financially from the publication of this manuscript, either now or in the future?
- Do you hold any stocks or shares in an organisation that may in any way gain or lose financially from the publication of this manuscript, either now or in the future?
- Do you hold or are you currently applying for any patents relating to the content of the manuscript?
- Have you received reimbursements, fees, funding, or salary from an organization that holds or has applied for patents relating to the content of the manuscript?
- Do you have any other financial competing interests?
- Do you have any non-financial competing interests in relation to this paper?

If you can answer no to all of the above, write 'I declare that I have no competing interests' below. If your reply is yes to any, please give details below.

I declare that I have no competing interests

I agree to the open peer review policy of the journal. I understand that my name will be included on my report to the authors and, if the manuscript is accepted for publication, my named report including any attachments I upload will be posted on the website along with the authors' responses. I agree for my report to be made available under an Open Access Creative Commons CC-BY license (http://creativecommons.org/licenses/by/4.0/). I understand that any comments which I do not wish to be included in my named report can be included as confidential comments to the editors, which will not be published.

I agree to the open peer review policy of the journal
