# Supplementary material for: ProphTools: general prioritization tools for heterogeneous biological networks
Source: Gigascience. 2017 Nov 24;6(12):1–8. doi: 10.1093/gigascience/gix111 (PMC5751048; doi:10.1093/gigascience/gix111)
Supplement: Reviewer-2-Report-(Original-Submission).pdf [file gix111_reviewer-2-report-(original-submission).pdf]

Reviewer Report

Title: ProphTools: General Prioritization Tools for Heterogeneous Biological Networks

Version: Original Submission **Date:** 7/3/2017

Reviewer name: Joseph J. Nalluri

Reviewer Comments to Author:

The authors have packaged their algorithm and previous work into a tool for usage by broader research community. Hence, while the algorithm and its efficacy is established, the usage of this tool for a generic applicability and its usability/user-friendly aspect can be improved.*All the line numbers mentioned below refer to the original manuscript's line numbers*Technical:1) Only because the authors have positioned ProphTools as a "general, ready to use", "can be used out-of-the-box" tool, it gives an impression that the tool is ready to be downloaded, plugged-and-played. However, in my estimation, this is not the case. For e.g.,a) Generally such tools would allow for the input to be a .txt or .csv file with minimal restrictions on the input type/nature and minimal preprocessing of the input. For e.g. Girvan-Newman's community detection command line tool, or GP-Dream network inference tools, etc. ProphTools "requires" the input to be in a .mat file which requires additional familiarity with either Matlab or Python and a significant amount of input-preprocessing (e.g. adjacency matrixes, precomputed matrices, required meta-data). What's a precomputed matrix? What should it contain?b) The "How to use" instructions can be described better, step-by-step from creating a .mat file and its necessary components and formats.c) "The generality of ProphTools prioritization requires you to provide some meta-data along with the adjacency matrices for the entities and the relations involved in your network configuration." - Where/when should the user provide this meta-data, because in the instructions on usage, the command is "prophtools prioritize --matfile network.mat --src 0 --dst 2 --query 1,2" and the input network.mat file does not have any meta-data as described in the "README.rst".2) In the motivation, the authors have emphasized in their writing, figures and example code about the application of ProphTools to heterogenous networks of atleast 3 sub-networks. However, in the case study, they have implemented ProphTools only 2 sub-networks (lncRNAs and diseases). It would certainly be more convincing with an application of atleast 3 subnetworks, especially since its written on line 105 "For instance, if a drug-target-disease network configuration is provided, ProphTools' ability to predict drug-disease relations can be estimated by performing a 5-fold LOO-CV test on such relation."3)The amount of preprocessing required (in addition to familiarity with Matlab/Python) is quite substantial and hence, if these tasks/datasets can be automated then it makes ProphTools more user-friendly and ready to use as an out-of-box utility.3) In general, this work lacks substantial motivation - on line 67, it says "ProphTools is based on an approach that has been proven useful in several prioritization applications, such as gene-disease prioritization[8] and drug repositioning[9]". The cited references are the authors' previous works. Hence, a broader literature review describing the diverse applicability and potency of the prioritization method is needed.4) The case study is good and certainly highlights the usability of the tool; however, the significant preprocessing performed here underscores my aforementioned concern of "ready-to-use and out-of-

the-box" ease of usage in #1.Non-technical:1) Typos can be corrected.- Line 30, "...as nodes in a graph, *whereas* weighted edges correspond to interactions or any type of relationship...". Should be "where" and not "whereas".- Line 43, "RANKS[2] performs node prioritization on some label *o* property by using kernelized score functions". "of" ?- Line 117, "systematically" has been misspelled as "sistematically".- Case study and not "Case of study".- Lines 257-261 repeat the information earlier stated and are not necessary.The authors have done a commendable job in making their code, data easily available, reproducible and abided by good programming standards of software engineering and code distribution.

Level of Interest

Please indicate how interesting you found the manuscript: An article of limited interest

Quality of Written English

Please indicate the quality of language in the manuscript: Needs some language corrections before being published

Declaration of Competing Interests

Please complete a declaration of competing interests, considering the following questions:

- Have you in the past five years received reimbursements, fees, funding, or salary from an organisation that may in any way gain or lose financially from the publication of this manuscript, either now or in the future?
- Do you hold any stocks or shares in an organisation that may in any way gain or lose financially from the publication of this manuscript, either now or in the future?
- Do you hold or are you currently applying for any patents relating to the content of the manuscript?
- Have you received reimbursements, fees, funding, or salary from an organization that holds or has applied for patents relating to the content of the manuscript?
- Do you have any other financial competing interests?
- Do you have any non-financial competing interests in relation to this paper?

If you can answer no to all of the above, write 'I declare that I have no competing interests' below. If your reply is yes to any, please give details below.

I declare that I have no competing interests

I agree to the open peer review policy of the journal. I understand that my name will be included on my report to the authors and, if the manuscript is accepted for publication, my named report including any attachments I upload will be posted on the website along with the authors' responses. I agree for my

report to be made available under an Open Access Creative Commons CC-BY license (http://creativecommons.org/licenses/by/4.0/). I understand that any comments which I do not wish to be included in my named report can be included as confidential comments to the editors, which will not be published.

I agree to the open peer review policy of the journal
